# High temporal resolution monitoring of illicit drug consumption across England via wastewater analysis

**DOI:** 10.1111/add.70398

**Published:** 2026-04-26

**Authors:** Helena Rapp‐Wright, Keng Tiong Ng, Derryn Grant, William Francis, Margarita White, Dimitris Evangelopoulos, Yuxing Liu, Konstantina Dimakopoulou, Sofia Zafeiratou, Dylan Wood, Chryshanthi Christy, Stav Friedman, Timothy W. Gant, Klea Katsouyanni, Leon P. Barron

**Affiliations:** ^1^ Medical Research Council (MRC) Centre for Environment and Health, Environmental Research Group, School of Public Health Imperial College London London UK; ^2^ National Institute for Health Research Health Protection Research Units (NIHR HPRU) in Environmental Exposures and Health Imperial College London London UK; ^3^ Department of Hygiene, Epidemiology and Medical Statistics, Medical School National and Kapodistrian University of Athens Athens Greece

**Keywords:** co‐administration, high‐throughput analysis, illicit drugs, ketamine, LC–MS/MS, spatio‐temporal trends, wastewater surveillance

## Abstract

**Aim:**

To monitor community‐level consumption of 20 illicit drugs across urban areas of England using wastewater‐based epidemiology (WBE) surveillance at high temporal resolution.

**Design, setting, cases:**

This study was conducted over a 12‐month period in 2022 sampling 24‐hour composite wastewater samples at 15 wastewater treatment plants (WWTPs) covering catchment population equivalents ranging from ~100 000 to >1 million. Analysis was conducted using rapid liquid chromatography–tandem mass spectrometry methods. The sampled WWTPs collectively covered 21% of the national population.

**Measurements:**

Primary data outcomes were the population‐normalised daily loads (PNLs) entering the WWTP, estimated population‐normalised consumption (both in mg/1000 people/day) and total mass (g/day) of 20 targeted illicit drugs and total mass in each catchment, quantified using suitable drug target residue markers in 1746 wastewater samples. Covariables included temporal indicators (e.g. public holidays, events) and regional factors. Presence, quantity and correlation of WBE‐derived drug use data were used to infer drug use patterns.

**Findings:**

Of the 20 illicit drugs investigated, 18 were detected in at least one sample. Cocaine exhibited the highest average daily PNL (2770 ± 829 mg/1000 people/day), followed by heroin (382 ± 248), ketamine (287 ± 183), amphetamine (272 ± 268), 3,4‐methylenedioxymethamphetamine (MDMA) (80 ± 57) and methamphetamine (60 ± 99) across 2022. When comparing PNLs to Sewage analysis CORe group—Europe (SCORE) and European Drugs Agency WBE data for 109 other WWTPs across Europe from March to May, 2022 cocaine and ketamine PNLs from sites in England were ranked statistically higher [cocaine: Wilcoxon rank‐sum test statistic (W) = 971, adjusted *P* = 0.000115; ketamine: W = 264, adjusted *P* = 0.0000389]. Importantly, seven English WWTPs recorded higher mean ketamine PNLs than any other European site over the same period in 2022. Temporal spikes in drug consumption aligned with public holidays and major events. A notable decrease in cocaine use coincided with a 3.7‐t UK seizure. Strong inter‐drug correlations were observed across catchments, particularly for benzoylecgonine/ketamine and benzoylecgonine/cocaethylene. Extrapolation to generate a representative national average consumption estimate is not recommended, as the WWTPs studied were mostly classified as urban areas and found not to be representative of the entire population of England.

**Conclusions:**

Wastewater analysis revealed widespread and temporally variable illicit drug use across England in 2022, with ketamine use exceeding European levels at multiple sites. The findings highlight wastewater‐based epidemiology's capacity to monitor drug use trends and identify community‐level impacts of interventions and events.

## INTRODUCTION

In 2022, 4907 deaths related to drug poisoning were registered in England and Wales, the highest number since 1993 [1]. The rate of deaths from drugs was highest for individuals aged 40–49 years. Almost half of drug deaths involved an opiate (*n* = 2261 deaths), mostly comprising heroin or morphine (*n* = 1256). In 2022, deaths associated with cocaine use rose for the 11th consecutive year (*n* = 857 deaths), and cocaine was consistently the second most used drug after cannabis [2]. Aside from drug‐related deaths, 9.2% of adults reported being frequent users of drugs in the year up to June 2022, which rose to 9.5% by March 2023, representing 3.1 million people [2]. Class A drugs (classified as the most dangerous drugs and therefore subject to the harshest penalties) were taken by approximately 1.1 million people aged 16–59 years [2]. Therefore, there are now escalating concerns of a public health crisis arising from adverse effects on human health and on social welfare [3, 4]. Taking the harm to health, costs of crime and wider impacts on society in total into account, the cost of tackling the drug problem in England and Wales is estimated at £19 billion annually [5]. This highlights the need for rapid surveillance approaches that can be used to identify and respond to this population‐wide crisis.

Monitoring drug consumption trends in near real‐time via wastewater can help with emerging reduction strategies as well as developing strategic actions [3, 6]. Wastewater‐based epidemiology (WBE) surveillance of illicit drug consumption has been successfully used for over 15 years at national and international levels [7, 8, 9], through the estimation of community‐scale consumption of drugs by analysing a pooled untreated water sample collected at a wastewater treatment plant (WWTP) [10, 11, 12]. This allows the investigation of drug‐use trends with quantitative data, compared with more traditional qualitative monitoring such as population surveys and police seizures [13]. In 2011, the Sewage analysis CORe group—Europe (SCORE), together with the European Union Drugs Agency (EUDA), ran its first international comparison study to measure the usage patterns of five drugs: amphetamine, cocaine, methamphetamine, 3,4‐methylenedioxymethamphetamine (MDMA) and Δ9‐tetrahydrocannabinol (cannabis). Several countries have demonstrated its applicability at national level [9, 14, 15]. Therefore, the value of WBE surveillance for drug misuse monitoring has been steadily growing as a useful tool for near real‐time trend analysis, including the monitoring of emerging compounds like performance‐enhancing substances at community levels [16, 17].

Performing routine monitoring of chemicals in wastewater at large scale with a high level of confidence to provide reliable temporal and spatial knowledge presents many challenges. Owing to the moderate polarity of most illicit drugs, the most common technique used for analysis is liquid chromatography coupled with tandem mass spectrometry (LC–MS/MS) [13], using triple‐quadrupole mass analysers for quantitation, due to their high sensitivity, and/or high resolution mass analysers for their selectivity for qualitative non‐target screening. Generally, relatively large sample volumes have been required to enable sufficiently sensitive measurements to be made in such complex samples, by performing time‐consuming extraction techniques such as solid‐phase extraction (SPE) using volumes of >100 mL [8, 13, 18]. Other challenges arise from sample collection for illicit drugs, in particular, such as low or intermittent sampling frequency, access to WWTPs at weekends, the availability of grab versus composite samples, the storage capacity for large numbers of samples, and analyte stability considerations at the WWTPs, in transit and at the laboratory [13, 19, 20, 21]. Recently, and mainly because of advances in LC–MS/MS sensitivity, new methodologies have focused on the identification and quantification of chemicals in smaller wastewater samples, including for pre‐concentration using SPE (e.g. 50 mL) [20] and especially where direct‐injection LC–MS/MS can be performed [22, 23, 24]. Our laboratory has successfully developed and applied rapid direct‐injection methods for >200 chemical residues found in wastewater and surface water, taking approximately 5 minutes [25, 26, 27, 28, 29], and including several illicit drugs, down to low/sub‐ng/L concentrations. However, despite the sensitivity of direct‐injection methods, some challenges remain, particularly for very potent drug substances, such as markers of (synthetic) opioids, which are present typically at very low concentrations to detect, even with the use of SPE. In addition, and for consideration during sampling, some also display some degree of instability, affecting reliable measurement [30]. Overall, however, direct‐injection LC–MS/MS methods represent an excellent solution for large‐scale programmes. Lastly, the move towards automated data analysis workflows is now even more critical to enable knowledge transfer to authorities in a timely manner.

In 2021, and in partnership with the UK Home Office, the National Crime Agency and the Defence Science and Technology Laboratory (Dstl), the UK Wastewater Analysis Programme (WWAP) was launched to monitor drug misuse across England using WBE surveillance. The aim of this first phase of work was to establish an in‐depth baseline of drug use in England across 15 prioritised locations measured at high temporal resolution (sampled at least once every 2 days). Our study had four objectives: objective 1, to quantitatively monitor 20 prioritised illicit drugs, their metabolites and cutting agents in 1746 wastewater samples across 2022 using a combination of direct‐injection LC–MS/MS and SPE LC–MS/MS methods; objective 2, to investigate consumption trends and correlations between drugs within and between catchments and in accordance with international WBE surveillance studies; objective 3, to identify any impact of public holidays, large events and law enforcement seizures on drug consumption; and objective 4, the extrapolation of data to more reliably estimate yearly consumption in each catchment. This article represents the most intensive study of illicit drug consumption using WBE in England to date, particularly to understand temporal variations in selected urban areas.

## METHODS

### Reagents, chemicals and consumables

LC–MS/MS grade methanol and acetonitrile, and analytical high‐performance liquid chromatography (HPLC) grade acetonitrile, methanol and formic acid were purchased from Fisher Scientific (Loughborough, UK). Millipore Milli‐Q‐water purification system at 18.3 MΩ (Millipore, Bedford, MA, USA) was used to supply ultrapure water. All reference standards and stable isotope‐labelled internal standards (SIL‐IS) were ≥98% purity and included 5‐aminoisotonitazene, 6‐acetylcodeine, amphetamine, benzocaine, benzoylecgonine (BZE), cocaethylene, cocaine, 6‐monoacetylmorphine (6‐MAM), ethylidene‐1,5‐dimethyl‐3,3‐diphenylpyrrolidine (EDDP), isotonitazene, ketamine, lidocaine, methadone, methamphetamine, MDMA (ecstasy), morphine, norketamine, phenacetin, procaine and tetramisole (levamisole) were acquired from Sigma‐Aldrich (Steinheim, Germany). SIL‐IS standards, including amphetamine‐d_6_, benzoylecgonine‐d_3_, cocaine‐d_3_, ketamine‐d_4_‐HCl, MDMA‐d_5_, morphine‐d_3_ and venlafaxine‐d_6_‐HCl were purchased from Sigma Aldrich. Lidocaine‐d_10_‐HCl was ordered from QMX (Essex, UK). Stocks were prepared in methanol at 1.0 or 0.1 mg/L and stored at −20°C in silanised amber vials. These were further diluted weekly in methanol to prepare multi‐compound working solutions, which were stored under the same conditions as the stock solutions.

### Sample collection

**FIGURE 1:**
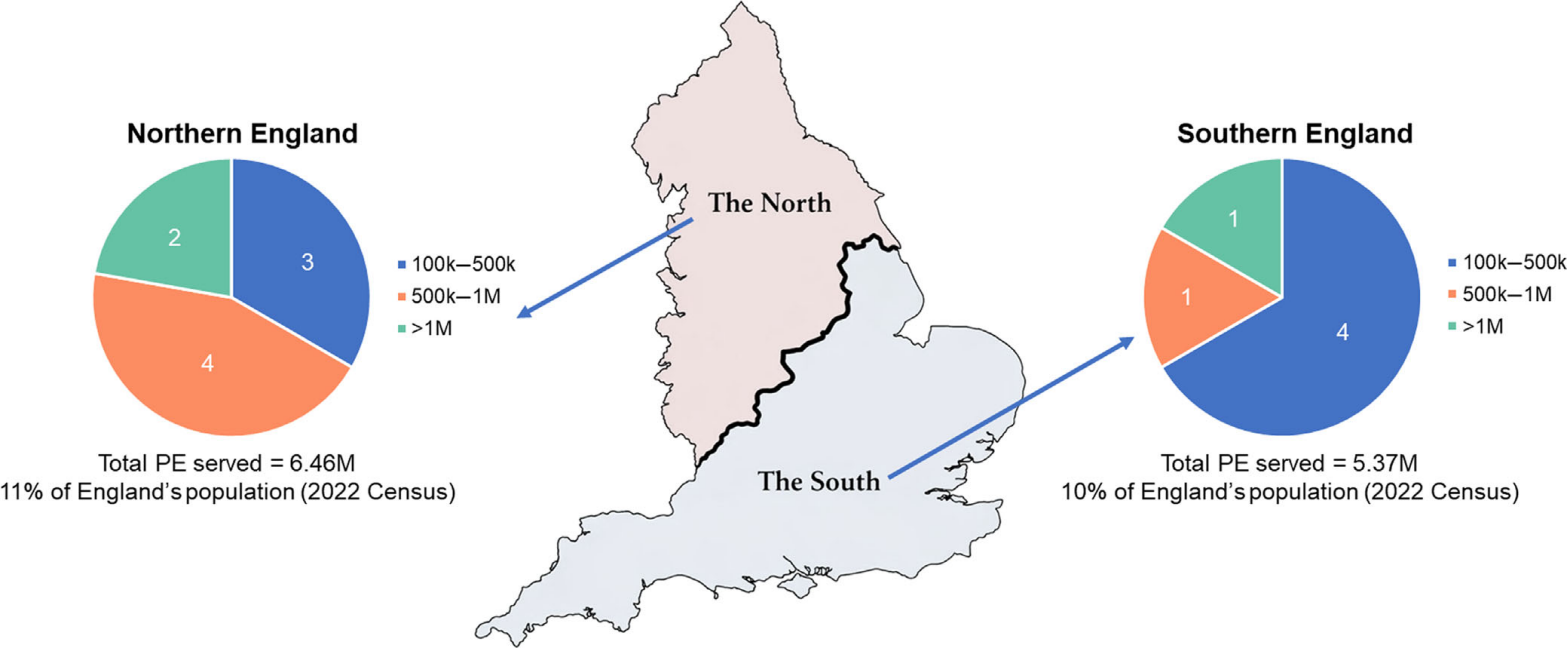
Map of England showing the division between the north and the south of the country, with pie charts showing the number of wastewater treatment plants (WWTPs) in each region and their classification by a banded scale of population equivalents (PEs).

To address objective 1, samples were collected from 15 different WWTPs spread across England with population equivalents (PEs) ranging from approximately 100 000 to 3 500 000 people, and then analysed using LC–MS/MS. Specific WWTP locations are not named, owing to confidentiality agreements, but are categorised into Northern and Southern regions of England, in accordance with the work of Dorling [31] (Figure 1). Sites were selected mainly based on geographical spread across the country as well as following UK Government priority to capture the largest combined possible PE, with a total estimated PE coverage of 11.8 million, equivalent to 21% of that of England. All sites provided daily influent flow measurements. Influent 24‐hour time‐proportional composite samples (at <60 minute frequency) were collected using refrigerated Glacier® portable water samplers set at 4°C (Teledyne ISCO, Lincoln, NE, USA) and located after the fine screen and before the primary clarifier. Nalgene bottles (Thermo Fisher Scientific, Rochester, NY, USA) were used to take 30‐mL samples in duplicate. Samples were collected at or above a frequency of 14 days per month, covering two separate daily samples for each day of the week. In some cases, WWTPs supplied samples every day, particularly in the earlier months, to ensure smooth onboarding and to establish a reliable baseline. Some sites (i.e. K–O) were brought on‐board later in the programme than others. Nine WWTPs were in place at the start of 2022 (i.e. A–F, I and J in January, and H in April). After collection the following day, samples were immediately stored at −20°C on site and in the dark, to minimise transformation and/or degradation of the compounds. The UK has a privatised water industry with nine separate water utility companies in England alone. More advanced sample preservation treatments on site (e.g. the addition of chemicals such as acids, stabilisers, etc.) were not possible owing to WWTP operator training levels and risk. Samples were stored, batched and shipped monthly to the laboratory within polystyrene boxes filled with ice packs and in 4°C cooled vans for a maximum transit time of 8 h. Upon reaching the laboratory in London, samples were stored at −20°C until analysis within 7 days.

### Sample preparation

For analysis with direct‐injection LC–MS/MS, 2‐mL centrifuge filters (Thermo Fisher Scientific) were used to prepare and store the samples, where 100 μL of a standard solution in methanol was added, including SIL‐IS, where needed, to a 900‐μL fixed volume of sample to obtain a final volume of 1 mL using positive displacement pipettes [27, 28]. Samples were then filtered using 0.2‐μm polytetrafluoroethylene (PTFE) Thermo Scientific™ 2‐mL non‐sterile centrifugal filters (Thermo Fisher Scientific) and then transferred to LC amber vials for analysis (Agilent Technologies, Santa Clara, CA, USA). Separate matrix‐matched calibration curves for each batch and WWTP site were prepared from 0 to 40 000 ng/L (*n* = 17) and SIL‐IS were added at a constant concentration of 500 ng/L for the quantification of nine prioritised compounds (see supporting information). For the remainder, background‐subtracted external matrix‐matched calibrations were used for quantification, as matching SIL‐IS were not available.

To achieve sufficient analytical sensitivity for relatively lower concentrations of 6‐MAM present in wastewater, SPE was used with HyperSep™ Retain PEP cartridges (200 mg, 6 mL; Thermo Fisher Scientific). Cartridges were conditioned with 4 mL of methanol and 4 mL of ultrapure water. Then, 15 mL of sample was loaded (with SIL‐IS added after thawing and before SPE), cartridges washed with 4 mL of methanol : water (5 : 95, v/v) solution and then dried under vacuum for approximately 20 minutes. Elution was performed in 4 mL of methanol. Extracts were then evaporated using N_2_ at 35°C and reconstituted in 150 μL of acetonitrile : methanol (1 : 1, v/v) : water (20:80, v/v). Finally, extracts were centrifuged and filtered using 0.2‐μm PTFE Single Step™ nano filter vials (Thames Restek, Saunderton, Buckinghamshire, UK). Calibration curves for 6‐MAM were prepared in ultrapure water again using SPE, ranging from 0 to 75 ng/L (*n* = 9) (pre‐extraction), using a constant concentration of 10 ng/L for the SIL‐IS analogue. In line with other studies showing the instability of this compound in wastewater [32, 33], only the most recent samples collected in the preceding 7 days before each shipment per site were analysed. Stability data for 6‐MAM and its SIL‐IS measured in a frozen UK wastewater matrix are shown in Figure S1.

### Instrumentation

Analysis was performed using a Shimadzu LCMS‐8060NX (Shimadzu Corporation, Kyoto, Japan) with a Raptor biphenyl analytical column (30 × 3.0 mm, 2.7‐μm particle size) fitted with a compatible guard column (5 × 3.0 mm, 2.7‐μm particle size) (Thames Restek). A sample injection volume of 10 μL was used, and mobile phases were 0.1% (v/v) formic acid in ultrapure water (A) and 0.1% (v/v) formic acid in acetonitrile : methanol (1 : 1, v/v) (B) at a flow rate of 0.5 mL/minute. Two different gradients were used for separation, one for the direct‐injection LC–MS/MS analysis and one for the 6‐MAM analysis alone. Both gradient elution profiles can be found in the supporting information. Multiple reaction monitoring (MRM) was performed in positive electrospray ionisation (ESI) mode only. At least two MRM transitions were used for confirmation, where the most intense transition was used for quantification purposes. For SIL‐IS, only one transition was used for quantification purposes. All MRM transitions (Tables S1 and S2) and the ESI conditions (Table S3) can be found in the supporting information. Performance data for both methods in matrix form are presented in Tables S4 and S5 and in accordance with the International Council for Harmonisation of Technical Requirements for Pharmaceuticals for Human Use (ICH) guidelines [34].

### Back‐calculation procedures for the estimation of drug consumption

To address objective 2, and for ease of direct comparison with EU‐wide monitoring data published by SCORE/EUDA, back‐calculations were performed as described by González‐Mariño *et al*. [11]. Daily influent flow data (m^3^/day) as well as the recorded PE were provided by each WWTP, which were then used to determine back‐calculated population‐normalised consumption from wastewater data. Flow data were used to calculate the total amount of a drug target residue (DTR) entering the WWTP in kg/day. DTRs were selected to represent each drug and are based on their stability in the wastewater network, low sorption to biosolids and where they give more specific detail on human consumption of a drug [35]. In many cases, these were primary metabolites, but sometimes these were the parent narcotic compound itself (e.g. MDMA and methamphetamine). Daily population‐normalised loads (PNLs) were calculated using the measured DTR concentrations following Equation S1. Given the scale of the analysis involved, only the aqueous fraction was measured, and suspended particulate matter (SPM) was removed by filtration prior to analysis. PNLs for all compounds were used for relative comparisons and correlations. Comparisons of measured PNLs at specific sites in England were examined with equivalent EUDA estimates for EU countries, using reported data for 109 WWTPs in 2022 (https://www.euda.europa.eu/publications/html/pods/waste-water-analysis_en).

To translate PNL data to the amount consumed by a population, correction factors for six drugs (Table 1) were employed to consider the average excretion rate, from all administration routes, the stability and the ratio of the molecular weight of the DTR versus the active ingredient, according to Equation S2. Consumption of cocaine was calculated using the measured concentrations of the BZE metabolite and 6‐MAM for heroin. PNLs for methadone and EDDP were not taken forward to estimate consumption owing to the higher measured biosolid sorption reported previously in the UK [40]. The molecular logP of all compounds investigated can be found in Table S6. Exfiltration was not included in the calculations here because of a lack of reliable data. All dates shown represent the day the autosampler began its 24‐h sampling period. The analytical method used in this work passed independent quality review in 2022 as part of the international laboratory assessment exercise conducted by SCORE [41]. Measured PNL data and reported consumption estimates refer to the 24‐hour period that the autosampler was active at the WWTP, allowing direct comparisons with other literature data to be made [11]. Accordingly, amphetamine loads consumption was estimated only from its PNL without correction from other sources, including the metabolism of methamphetamine, direct disposal or medicinal use (the latter of which was found to be very low; for the largest WWTP at site F, see Figure S6). Similarly, no correction for methamphetamine was made for any other sources, including direct disposal.

**TABLE 1:** Drug target residues (DTRs) and correction factors used for back‐calculating community consumption of detected illicit/abused drugs in wastewater.

Compound name	DTR	Correction factor	Reference
Cocaine	BZE	3.59	[11, 36]
MDMA	MDMA	4.40	[11]
Amphetamine	Amphetamine	3.30	[36, 37]
Methamphetamine	Methamphetamine	2.44	[11]
Ketamine	Ketamine	3.30	[36, 37]
Heroin	6‐MAM	86.9	[36, 38, 39]

*Note*: Back‐calculated data were not used for comparisons with European Union Drugs Agency (EUDA)/Sewage analysis CORe group—Europe (SCORE) data sets, which used population‐normalised daily loads (PNLs) alone.

### Data analysis

Microsoft Excel 2010 (v16.48; Microsoft, Redmond, WA, USA) and RStudio (v2023.06.1; RStudio, Boston, MA, USA) running R 4.3.1 were used for data evaluation and statistical analysis. The associations between daily illicit drug PNLs sampled across the 15 WWTPs were investigated using linear mixed‐effects models, with a random intercept included for repeated measures within each of the WWTP sites. For 10 of the 20 drugs investigated, 50% of samples either provided no data or were recorded as zero and these drugs were removed from the mixed‐effects model analysis. Spearman correlations between drugs were investigated using PNL data; the interpretation of the coefficients of determination (*R* ^2^) is presented in Table S7. Outliers were isolated for deeper study (e.g. to examine coincidence with particular events, weekends or public holidays, under objective 3) and were defined as observations greater than the 75th percentile plus 1.5‐fold the interquartile range. Extreme outliers were defined as greater than the 75th percentile plus 3.0‐fold the interquartile range.

Bank holidays in England and Wales (https://www.gov.uk/bank-holidays) were used for temporal assessments, where if on a Friday or Monday, this was considered part of the bank holiday weekend. Student's unpaired *t*‐tests were performed to assess statistically significant differences between bank holidays and regular weekends, as well as between weekends and weekdays. To compare PNL values between England and other European SCORE sites, weekly mean concentrations were calculated for each site by averaging daily measurements from Monday to Sunday during the SCORE sampling period. This aggregation yielded one independent observation per site. Also, to address objective 2, sites were classified into two regions: England; and other European sites participating in the SCORE network. For each drug (i.e. amphetamine, cocaine, ketamine, MDMA and methamphetamine), weekly mean concentrations were compared between regions using Wilcoxon rank‐sum tests, as the data were not normally distributed. Statistical tests were conducted independently for each drug. To account for multiple comparisons across the five drugs, *P*‐values were adjusted using the Benjamini–Hochberg false discovery rate (FDR) correction. Statistical significance was defined as an adjusted *P*‐value of <0.05.

To address objective 4, and to assess the representativeness of the sites chosen across England, data at the Lower Layer Super Output Area (LSOA) level were collected from the official census and labour market statistics website using 2021 census data (www.nomisweb.co.uk). LSOAs are administrative spatial units in England and are used as the standard geography for the collection and dissemination of small‐area statistics. The median LSOA population was 1185. All WWTPs were mapped and the comparative distribution of variables, including population size/density and urbanicity, for the catchment area of each WWTP was examined by aggregating the relevant LSOA data. The percentage of LSOAs in England covered by the sampled WWTPs (*n* = 15) and percentage of LSOAs where WWTPs were not sampled (i.e. *n* = 1419 WWTPs) were calculated (Table S8). The proportions of urbanicity of each of these LSOA groupings were also compared (Table S9). A two‐proportion *Z*‐test was used to assess the statistical significance of the difference in the proportions at the nominal statistical level. Owing to the very large number of observations that could detect small differences as statistically significant, we further assessed representativeness empirically by the magnitude of imbalance in different levels of urbanicity.

## RESULTS AND DISCUSSION

### Illicit drug occurrence summary

A total of 1746 samples were analysed across all sites in 2022, representing 360 separate days. Eighteen out of 20 target analytes were detected and quantified. Eight substances were detected in every sample (BZE, cocaethylene, cocaine, EDDP, ketamine, lidocaine, methadone and morphine), and were also quantifiable at relatively higher concentrations in comparison with all other substances. For ease of comparison across English sites, but also to compare with those included in the EUDA/SCORE annual comparison wastewater monitoring programme, PNL data were used rather than back‐calculated consumption data, to minimise uncertainty, particularly in relation to the excretion factor.

**FIGURE 2:**
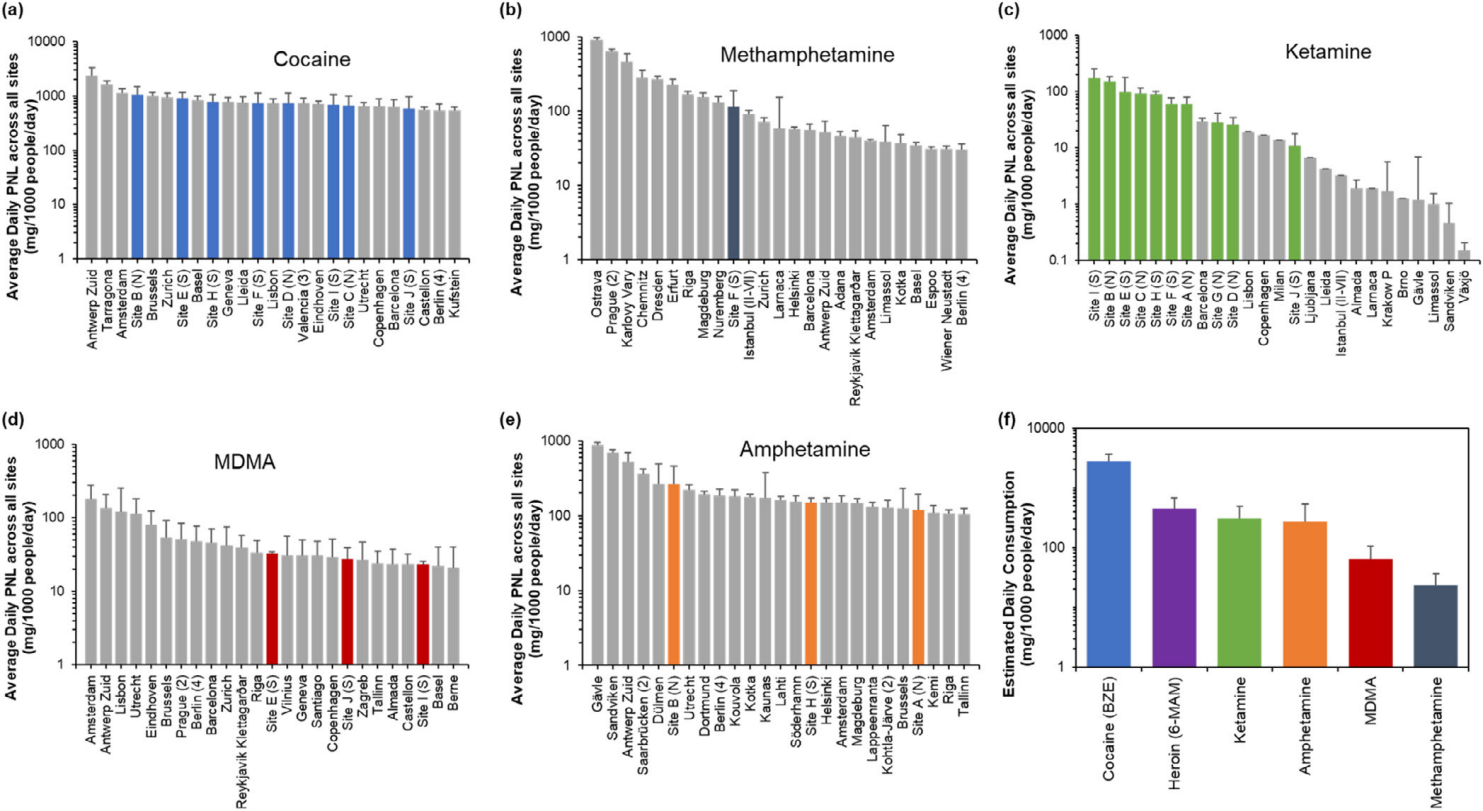
Time‐synchronised average population‐normalised daily load (PNL) data in mg/1000 people/day from English sites A–O monitored in this study: (a) blue = cocaine (benzoylecgonine, BZE); (b) dark blue = methamphetamine; (c) green = ketamine; (d) red = 3,4‐methylenedioxymethamphetamine (MDMA); and (e) orange = amphetamine; and in comparison wiother European catchments (in grey) reported as part of the European Union Drugs Agency (EUDA) wastewater‐based epidemiology (WBE) programme for the period 9 March–4 May 2022. Cities with multiple wastewater treatment plants (WWTPs) have the number of WWTPs monitored listed in parenthesis. (f) Average estimated consumption (mg/1000 people/day) across all sites using a logarithm scale, where error bars represent the standard deviation. Note: no EUDA data were reported for 6‐MAM in 2022 for comparison purposes, and so were excluded. Only *n* = 10 WWTPs had samples across the EUDA WBE sampling period. Bracketed letters indicate regional classification, where (N) corresponds to the north of England and (S) to the south of England.

First, and across all sites in England only, the PNL was relatively consistent and was highest for BZE (average 773 ± 231 mg/1000 people/day), in comparison with the other substances monitored (in descending order: BZE, cocaine, morphine, ketamine, amphetamine, lidocaine, EDDP, methamphetamine, MDMA, methadone, cocaethylene, norketamine, levamisole, 6‐acetylcodeine, benzocaine, 6‐MAM, phenacetin and procaine). Regarding EUDA/SCORE data, the daily average PNL data for 24 countries (ranging from 27 to 109 cities, depending on the compound) were reported for 2022, and for five substances included in this study (amphetamine, cocaine, ketamine, MDMA and methamphetamine). The most notable finding was for ketamine. When using data aligned with the same time period as the SCORE annual international monitoring programme (Figure 2), PNLs for seven catchments were higher, on average, than those of all other international WWTP catchments, highlighting the significant prevalence of ketamine across England in 2022. As sampling at five sites (i.e. K–O) were not yet active during this specific aligned time frame, a similar comparison of the full‐year data set is shown for all 15 sites in Figure S2, although these are less directly comparable.

Again, when aligning time frames with the EUDA/SCORE programme, cocaine PNLs were high for eight English WWTP catchments (i.e. within the highest 25 sites). For (meth)amphetamine compounds, the PNL for one WWTP catchment in England was considered relatively high, and PNLs for three WWTPs were considered relatively high for MDMA and amphetamine. Using Wilcoxon rank‐sum tests to compare sites in England with other European sites participating in the SCORE network over the same sampling period, cocaine concentrations differed significantly between regions (i.e. *P* < 0.05), with higher weekly mean values observed in England than in the other European SCORE sites (Wilcoxon rank‐sum statistic, *W* = 971, adjusted *P* = 0.000115). Ketamine concentrations also showed a highly significant difference (*W* = 264, adjusted *P* = 0.0000389), again with higher concentrations recorded in England. In contrast, no statistically significant differences were observed for amphetamine (adjusted *P* = 0.949), MDMA (adjusted *P* = 0.0642) or methamphetamine (adjusted *P* = 0.71). Although MDMA exhibited a nominal difference prior to adjustment, this effect did not remain significant after correction for multiple comparisons. Overall, these findings indicate pronounced regional differences in the weekly mean concentrations of cocaine and ketamine, whereas the concentrations of amphetamine, MDMA and methamphetamine were broadly comparable between England and other European EUDA/SCORE sites. When all data were included, the cocaine and ketamine PNLs were significantly higher in England (*W* = 143, adjusted *P* = 0.00000120, and *W* = 20, adjusted *P* = 0.00000262, respectively), whereas no significant differences were observed for the remaining compounds.

Low frequencies of detection across the samples were observed for some compounds, such as phenacetin, procaine and benzocaine (2%, 5% and 6%, respectively, across all sites). These are all local anaesthetics used in medicine, but have also been reported as adulterants for cocaine owing to their low cost, wide availability and that they can mimic the anaesthetic effect of cocaine [42, 43]. However, lidocaine, another cocaine adulterant, was quantifiable in all samples, ranging from 10 ng/L (site G) to 2576 ng/L (site I). This compound is also widely used through prescription to treat mouth ulcers and sore throats, with the National Health Service (NHS) English Prescribing Datasets (EPD) indicating 6275 kg of lidocaine having been prescribed in England in 2022 (https://opendata.nhsbsa.net/dataset/english-prescribing-data-epd). This was calculated by summing the mass of lidocaine in all lidocaine‐containing products across all sites for each month in 2022. Other compounds such as 6‐acetylmorphine, levamisole and norketamine were detected at moderate frequencies (46%, 35% and 19%, respectively), with generally a low number of samples containing quantifiable concentrations.

Aside from heroin or its metabolite 6‐MAM, two other new psychoactive substance (NPS) synthetic opioid compounds, isotonitazene, and its metabolite, 5‐aminoisonitazene, were not detected across any site or sample. To date, there are no studies confirming the presence of these specific nitazene compounds in wastewater, but another analogue, protonitazene, was recently detected in two cities in the USA with a frequency of detection of only 8% [44]. Until March 2020, there had not been any reports in Europe of acute non‐fatal toxicity caused by isotonitazene in patients within hospital emergency departments [45, 46]. However, there has been an increase in presentations to hospital emergency departments in the UK since 2021 with acute opioid toxicity [45], and blood of postmortem cases related to potential heroin use throughout London and southeast England were screened, where isotonitazene has been regularly detected [47]. However, the routine detection and monitoring of these substances in wastewater would likely require much higher method sensitivity, given their high potency, where the instrumental limit of detection (LOD) by direct injection using wastewater matrix in this study is approximately 10 ng/L. In addition, a recent study using direct‐injection LC–MS/MS for NPS opioids showed that isotonitazene suffered the worst matrix effect in wastewater out of all compounds tested [48]. Preliminary efforts to detect these substances in wastewater samples using the concentrative SPE protocol for 6‐MAM also failed. The instability of these compounds in wastewater is not well understood and should be studied in more detail, like other NPSs, especially where delays exist between sampling and analysis [49].

### Trends in illicit drug consumption at high temporal resolution

Temporal trends were investigated across all sites and for all drugs in two ways, including overall trends across the whole year (using a rolling average of consumption) and an examination of drug consumption by day of the week.

**FIGURE 3:**
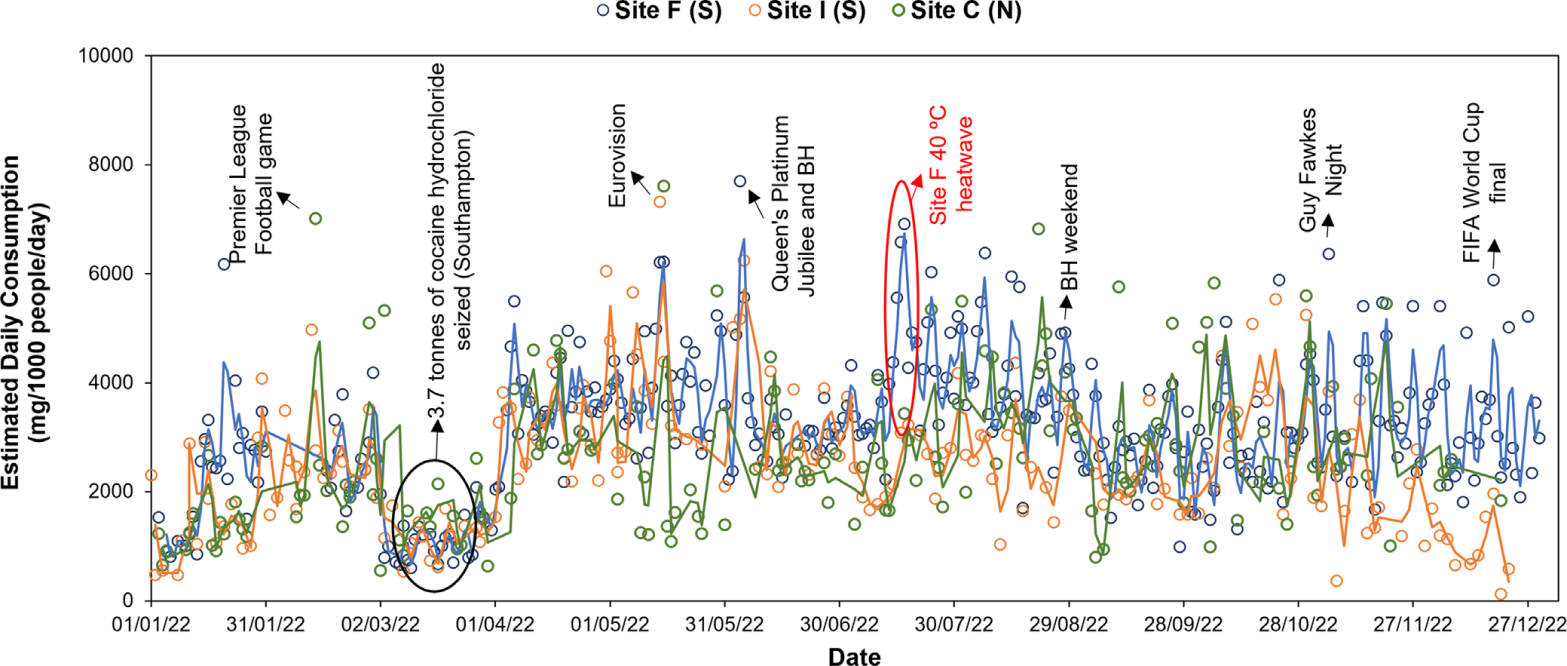
Time series rolling average plot of cocaine (benzoylecgonine, BZE) population‐normalised daily consumption in three sites per day (in mg/1000 people/day) for all samples analysed and by the full population equivalent (PE) served by the wastewater treatment plant (WWTP). The arrows highlight different events across the year. Bracketed letters indicate regional classification, where (N) corresponds to the north of England and (S) to the south of England.

First, and across the year, the overall estimated population‐normalised daily consumption (in mg/1000 people/day) varied markedly between WWTP catchments. Cocaine had the highest average per capita consumption estimate across all sites (2770 ± 829 mg/1000 people/day), followed (in descending order) by heroin (382 ± 248 mg/1000 people/day), ketamine (287 ± 183 mg/1000 people/day), amphetamine (272 ± 268 mg/1000 people/day), MDMA (80 ± 57 mg/1000 people/day) and methamphetamine (60 ± 99 mg/1000 people/day) (Figure S3). Consumption data for all sites are shown for all drugs in daily trends and quarterly intervals in Figures S4 and S5. Trends in amphetamine, ketamine, MDMA and methamphetamine consumption were relatively stable across 2022, but notable variation in consumption existed for heroin and cocaine across the year. Heroin consumption decreased in May and was followed by a slow but steady return by the end of the year. Though all the contributing factors for this remain unclear, this time frame did coincide with the announcement of the ban on the cultivation of opium poppy in Afghanistan (April 2022), which up to that time had represented the leading poppy producer, supplying 80% of heroin demand worldwide [50]. Overall, cocaine consumption was significantly higher in Q2 and Q3 than in Q1 and Q4 (Figure S5). A noticeable drop in cocaine consumption was observed across sites in March 2022 when compared with other months (Figure 3). All sites containing data in both months (*n* = 8) showed a decrease from February to March, with the percentage decrease ranging from 14% to 74%. A seizure of 3.7 tonnes of cocaine hydrochloride occurred at Southampton Docks on 17 March 2022, which was one of the largest in recent UK history [51]. All eight sites were back to average PNL levels in April 2022, except sites B and D, which recovered during April–May. The potential impact that this particular seizure may have had on cocaine consumption can be observed in Figure 3 for three example catchments located approximately 200 km apart and with a combined PE of approximately 5 316 061 (sites C, F and I). This finding demonstrates the power of WBE to understand the multi‐catchment impacts of seizures and the resilience of illicit drug supply chains. Further investigation should be conducted regarding the scale of seizures that affect consumption at both the local and the national level and over what time frame.

**FIGURE 4:**
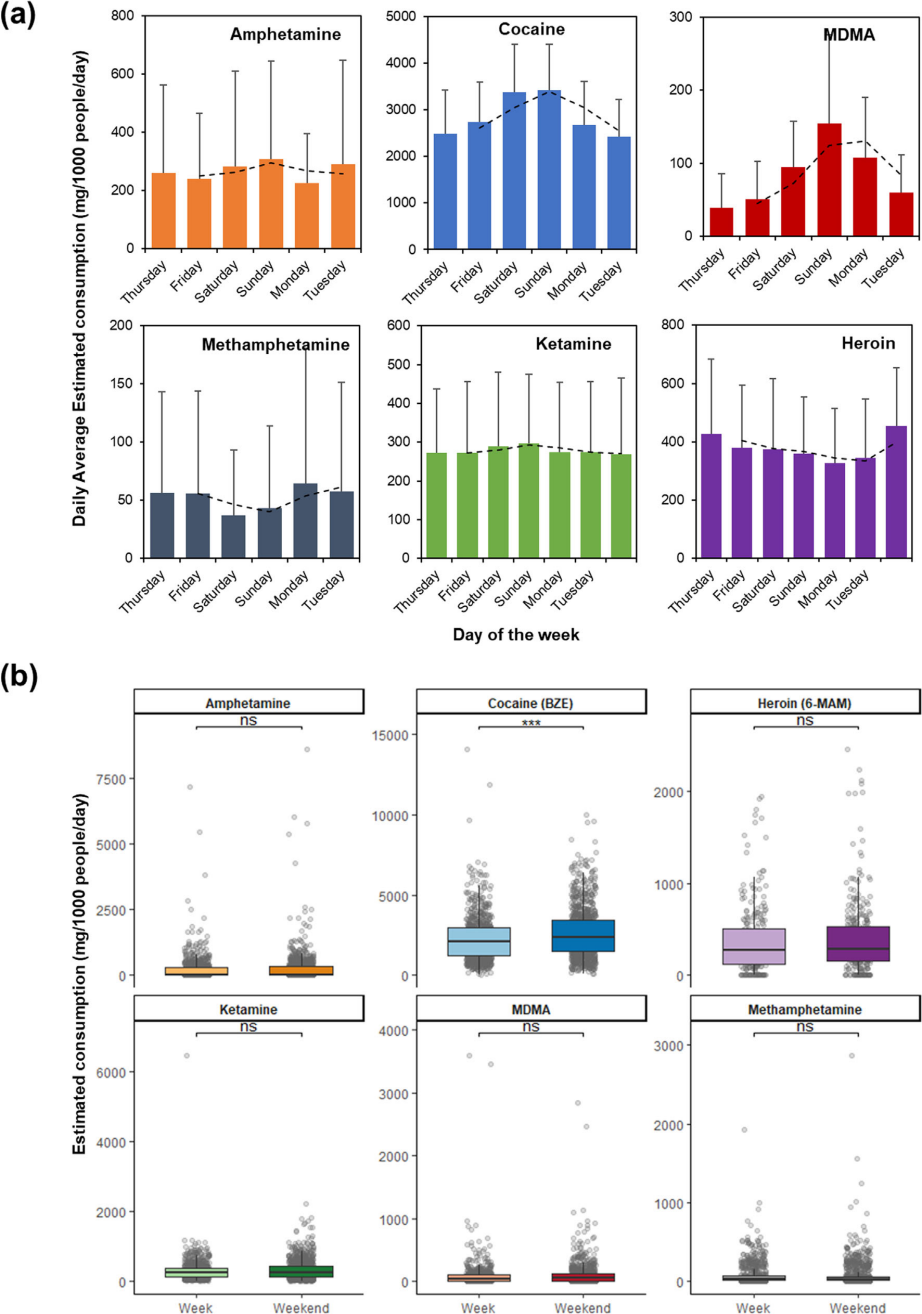
(a) Daily average of estimated consumption of all drugs across all sites per day of the week (mg/1000 people/day), where error bars represent the standard deviation and the trendline is the moving average fit (*n* = 2). All dates shown represent the day the autosampler began its 24‐h sampling period; therefore, consumption may reflect usage from the previous day(s). (b) Box and whisker plots of estimated consumption of drugs of all wastewater treatment plant (WWTP) catchments per day per 1000 people (in mg/1000 people/day) for all samples analysed in this study classified by week (Tuesday–Thursday, inclusive) and weekend (Friday–Monday, inclusive). Statistical comparisons between week and weekend values were performed using unpaired Student's *t*‐tests; ns = not significant (*P* > 0.05); *P ≤ 0.05; **P ≤ 0.01; ***P ≤ 0.001.

Drug consumption by day of the week across all sites (Figure 4) showed a clear recreational use trend at weekends for cocaine and MDMA (11 sites each), with marker excretion peaking mainly on Sundays. However, for the rest of the drugs investigated, the patterns were more mixed or remained steadier across the week across all sites. Ketamine consumption showed an obvious increase in only four catchments over the weekend, even for those catchments with overall high ketamine consumption levels. The available NHS data show that licit ketamine mass prescribed nationally decreased substantially across the years 2020–2024, and that the estimates for total daily mass consumed (kg/day) derived from wastewater measurements were several orders of magnitude higher (for site F, the largest WWTP studied, see Figure S6). Overall, methamphetamine consumption was small for most sites and showed no major recreational use trends over the weekends. However, consumption across the week in sites F and O were approximately 11‐ and 15‐fold higher than the other sites, respectively. Recent research in the UK has shown that methamphetamine along with ketamine, gamma hydroxybutyrate/gamma butyrolactone and mephedrone have been commonly used by men who have sexual contact with men (MSM) for ‘chemsex’, particularly in the south‐east of England [52, 53], which was partly supported by results from our WBE study locations in this region. Site F showed low recreational weekend use, while site O did not have any samples for Saturdays and Sundays and so comparisons could not be made. Higher weekend use of amphetamine was not evident in locations with higher overall consumption, including for site B, where levels were fivefold higher than the average of the other catchments.

### Impact of large‐scale public events and holidays on drug consumption

One of the main advantages of this intensive temporal monitoring approach was the ability to retrospectively identify other dates (and across WWTP catchments) where drug use coincided with major events. Daily consumption data outliers were first shortlisted across all sites and all drugs (with a total of 9193 measurements). On top of any variation observed across a typical week (e.g. from recreational use), a total of 369 outliers were obtained (4% of total data), of which 147 were considered extreme (1.6% of total data). Most of the extreme outliers occurred towards the end of the year in November (*n* = 36) and December (*n* = 24).

In general, elevated consumption was observed over bank holidays that, in the UK, occur on Fridays (*n* = 2) and mainly on Mondays (*n* = 11). Comparing consumption over bank holiday weekends with consumption over regular weekends using estimated consumption data across all sites (Fridays–Mondays, inclusive, in both cases), cocaine use on both the Fridays and Mondays of bank holidays was significantly higher (*P* = 0.021 and *P* = 0.020, respectively) when compared with regular Fridays and Mondays (approx. 21% higher on average). Comparison of all Saturday and Sunday PNL trends revealed no significant differences from regular weekends. MDMA showed a higher consumption on Fridays, Saturdays and Mondays over bank holidays (*P* = 0.0188, *P* = 0.0453 and *P* = 0.0016, respectively). For the remaining compounds monitored, no significant differences were observed (Figure S7). Furthermore, several extreme outliers for different drugs were found during the same day within the bank holiday weekend (e.g. 1 May for ketamine, *n* = 1, site I, and for MDMA, *n* = 3, sites D, H and J). Extreme outliers for the consumption of cocaine (14 072 mg/1000 people/day, site A, 2 August) and amphetamine (7165 mg/1000 people/day, site B, 1 November) were, in both instances, measured on a Tuesday, the day after the preceding bank holiday Monday. The latter instance was directly after Hallowe'en. In total, six sites had sample data for Hallowe'en itself. Two sites presented outliers for amphetamine, one site for cocaine, ketamine and methamphetamine, and three sites for MDMA. Previous WBE studies have also targeted other dates in the year that were likely to be correlated with drug consumption, such as New Year's Eve [54]. On 1 January 2022, only one site had WBE data on the day showing outliers for MDMA and methamphetamine consumption.

Other large events were also investigated, such as the dates coinciding with the FIFA Football World Cup in Qatar, held between 20 November and 18 December 2022. A total of 68 outliers were obtained in that period, of which 32 were extreme outliers. As an example, during this period the England national team played five games that all presented at least one consumption outlier across six sites with WBE data (F, A, D, G, N and M). Most outliers occurred on 4 December 2022 (Sunday), and were for cocaine (consumption of 6965 mg/1000 people/day, site D), amphetamine (307 mg/1000 people/day, site F), methamphetamine (51 mg/1000 people/day, site M), MDMA (526 mg/1000 people/day, site F) and ketamine (1792 mg/1000 people/day, site D). However, the elevated consumption on these dates may also be associated with regular weekend recreational usage. The consumption of drugs coincided with other recognisable events, such as the Eurovision Song Contest final (Saturday, 14 May 2022), for which WBE data were available for five locations, with two sites recording outliers for cocaine and one site recording an outlier for methamphetamine. On the following day, and from six sites with WBE data, three presented outliers for cocaine and MDMA, and two sites presented outliers for ketamine and methamphetamine. During the national Queen's Jubilee celebrations (2–5 June 2022), with a bank holiday on the 3 June 2022, some outliers were observed within catchments A, B, F and I, with populations ranging from approximately 286 000 to 3 380 000, but not necessarily all together for all substances. One of five catchments with WBE data presented an outlier for ketamine on 3 June 2022, one of four catchments presented outliers for cocaine and ketamine and two of four catchments presented outliers for MDMA on 4 June 2022, and one of five catchments presented outliers for cocaine and methamphetamine and three of five catchments presented outliers for MDMA on 5 June 2022.

Isolated events that occurred within single catchments were also detected. Site C presented high cocaine consumption the day after a major football game was held in the local area in February. However, the most obvious region‐specific of these was the high consumption of cocaine on 16–17 July 2022 and MDMA on the 17 July 2022 (site F), which coincided with one of the strongest UK heatwaves, which also represented a WWTP catchment with a high PE (>1 million). An elevated risk of drug‐related overdose deaths has been shown to exist during elevated ambient temperatures [55, 56]. Therefore, this suggests that WBE could potentially be used to further monitor and understand temperature‐driven changes in community consumption behaviour during acute atypically hot periods, but also across the hotter seasons in general.

Overall, this approach highlighted the value in high temporal resolution WBE in both catchment‐specific situations and for national events, to ascertain when and where interventions may be required.

### Correlation between drugs

**FIGURE 5:**
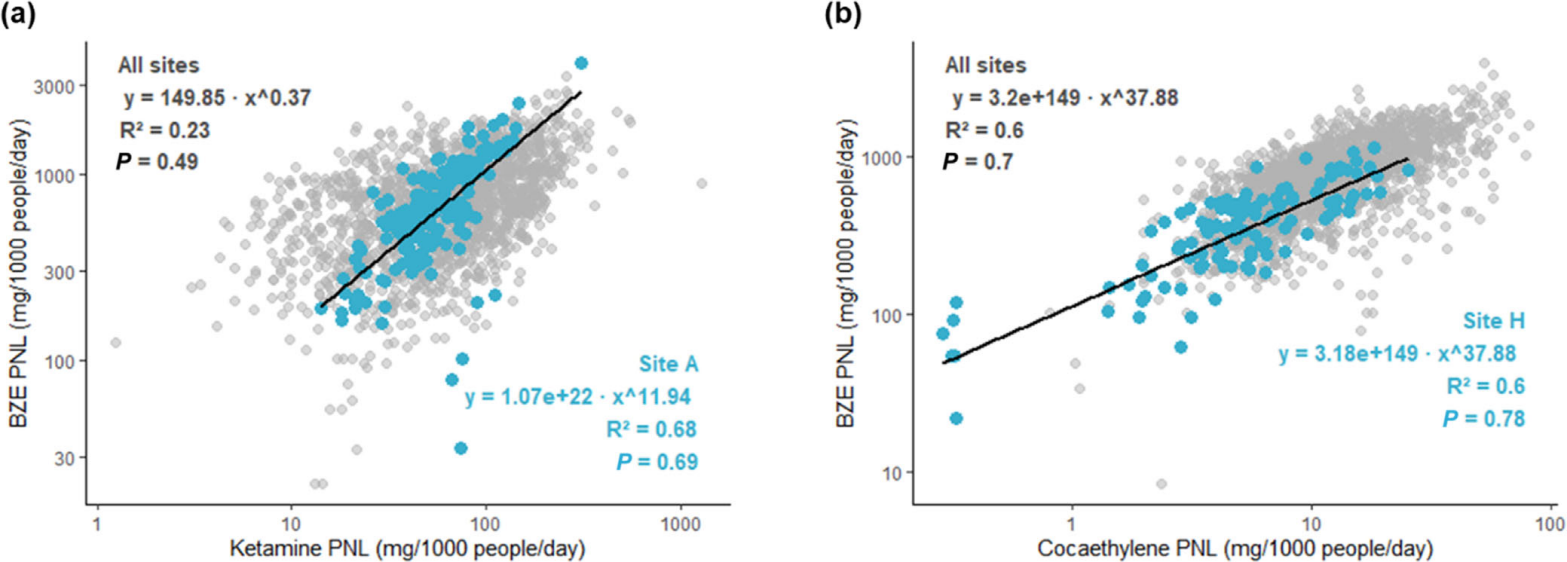
Scatter plot showing the correlations using population‐normalised daily load (PNL) data (mg/1000 people/day) across all sites and all dates (light grey) and for one selected site (highlighted in blue) in 2022 in logarithmic scales for: (a) site A using cocaine (as benzoylecgonine, BZE) and ketamine; and (b) site H using cocaine (as BZE) and cocaethylene. A linear regression model was fitted, and the resulting regression equation, coefficient of determination (*R* ^2^) and Spearman correlation (ρ) are annotated on the figure.

Paired PNL data between drugs across all sites were investigated. Figure 5 shows that it was critical to consider each site individually, rather than examining the data as a whole, with several individual sites showing high correlations between selected drug pairs at particular locations. Some of these pairings were, as expected, parent–metabolite pairs, including methadone and EDDP, and cocaine and BZE (Figure S8). In addition, some compound comparisons suffered from low detection frequency, such as the adulterants procaine, benzocaine and levamisole. Correlations between BZE and ketamine PNLs were generally weak across all catchments (*R* ^2^ = 0.23, Figure 5a) and their detection frequencies were very high (Table S9). However, when correlating data at single WWTP level, and without any further data treatment, medium to strong correlations (defined as Table S7) between these two substances were observed in six catchments (e.g. strong correlation at site A, *R* ^2^ = 0.68, shown in Figure 5a, and shown for all sites in Figure S9). Although co‐administration by the same individual could not be determined using this WBE approach, the polydrug ‘CK’ contains cocaine and ketamine, and it is quite a common ‘party’ drug not only in England but globally. On top of their individual risks [57], including their addictive properties [58, 59], the co‐consumption of these two drugs has significant and unpredictable health risks, including increased cardiovascular stress and respiratory depression, which can lead to sudden death, as well as to violent behaviour and psychotic reactions [60].

Medium to strong correlations were also observed between BZE and cocaethylene for nine sites (e.g. site H, *R* ^2^ = 0.60, Figure 5b, and across all sites, *R* ^2^ = 0.60). Cocaethylene indicates the co‐administration of cocaine with alcohol, which has been observed to be higher during the weekends [61]. Such co‐consumption has been linked to increased cardiovascular health risks [62, 63] and mental health‐related complications [64, 65]. In the absence of ethyl sulphate WBE data here (as a specific marker of alcohol use alone), the cocaethylene/BZE PNL ratio was investigated and was also found to be significantly higher during the weekends (Figure S10a), with individual days of the week showing a moderate U‐shape (Figure S10b). This indicates the consumption of proportionally more alcohol than cocaine as part of higher recreational use during the weekends. This trend was observed in most individual catchments, with the exception of four catchments (sites J, M, N and O) that had no statistically significant differences between either ratios or daily PNL data for cocaethylene and BZE between weekdays and weekends, indicating that the consumption of both substances was steady and proportional across the whole week for these four sites. In addition, of these four sites, site J indicated averages that were quite similar (Figure S11), suggesting that this catchment had a particularly steady co‐consumption of cocaine and alcohol across the whole week throughout 2022.

To more reliably examine the associations across catchments, those compounds where detection frequency was <50% (i.e. predominantly zero values for PNL) were removed (Table S10). The fact that a different number of samples exist and the observations are nested per WWTP, required adjustment for each WWTP by implementing a mixed‐effects regression model with a random intercept. Table S11 shows the resulting associations between drugs accounting for the dependence of observations within the 15 sampling sites. All the coefficients were statistically significant except between MDMA and lidocaine. To interpret the magnitude of the association, each coefficient denotes the change in the dependent variable per one PNL change in the independent variable. As examples, per one PNL unit increase in BZE (i.e. 1 mg/1000 people/day) there is an increase of 0.016 PNL units in cocaethylene, per one unit increase in cocaethylene there is a 24.942 unit increase in BZE, per one unit PNL increase in ketamine there is an associated increase of 1.503 PNL units in cocaine, and per one unit increase in cocaine there is a 0.123 unit increase in ketamine. The associations are generally significant because of the large overall number of samples (Table S12). While their magnitude can be described using the coefficients from the model, future work could focus on extending the number of sites and samples used to get more geographical and time‐period (e.g. seasonal or weekday–weekend) representation.

### Estimation of the daily mass of drug consumed across catchments

**FIGURE 6:**
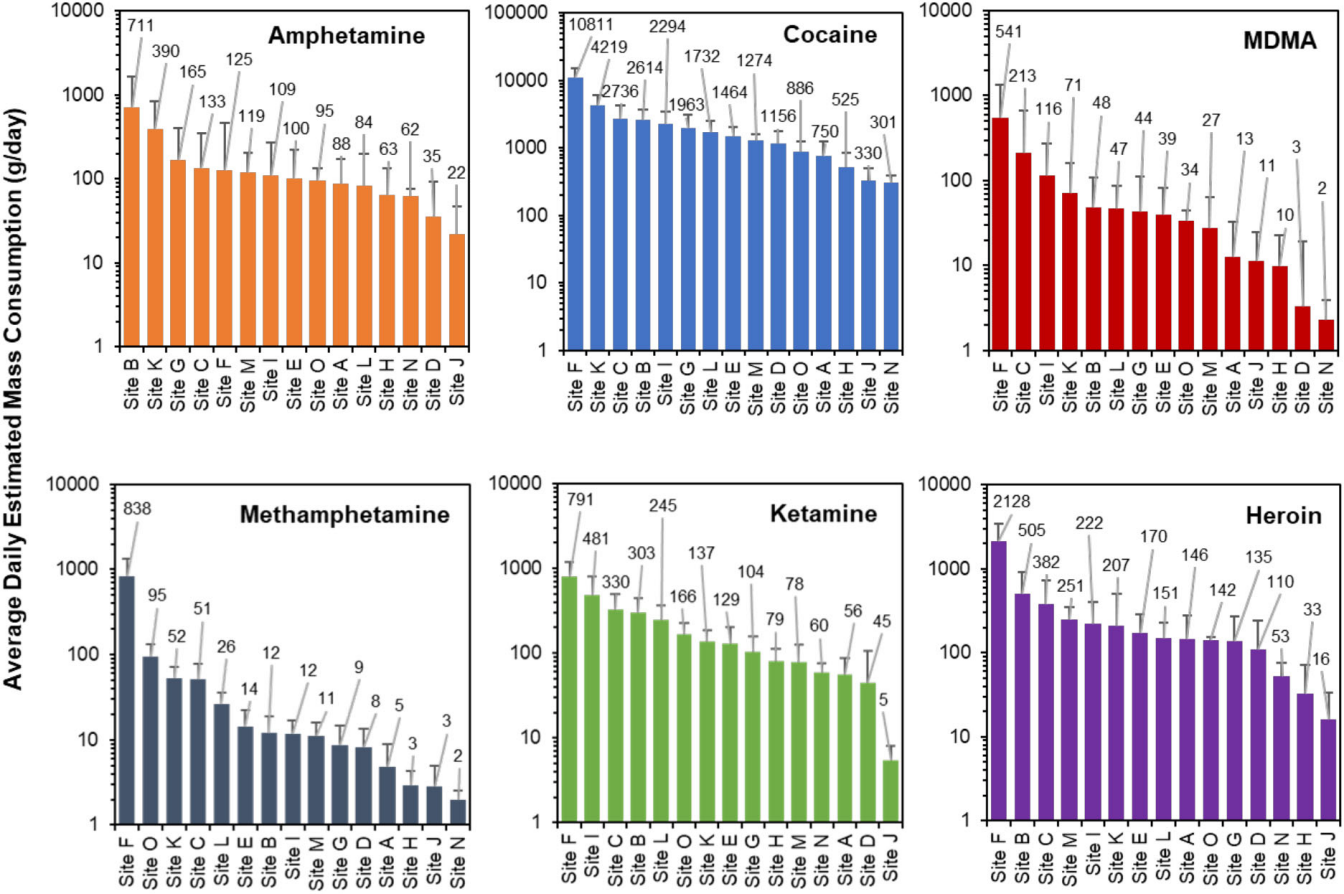
Daily mass of illicit drug consumed per wastewater treatment plant (WWTP) catchment area, where error bars represent the standard deviation across all measurements from each site.

**FIGURE 7:**
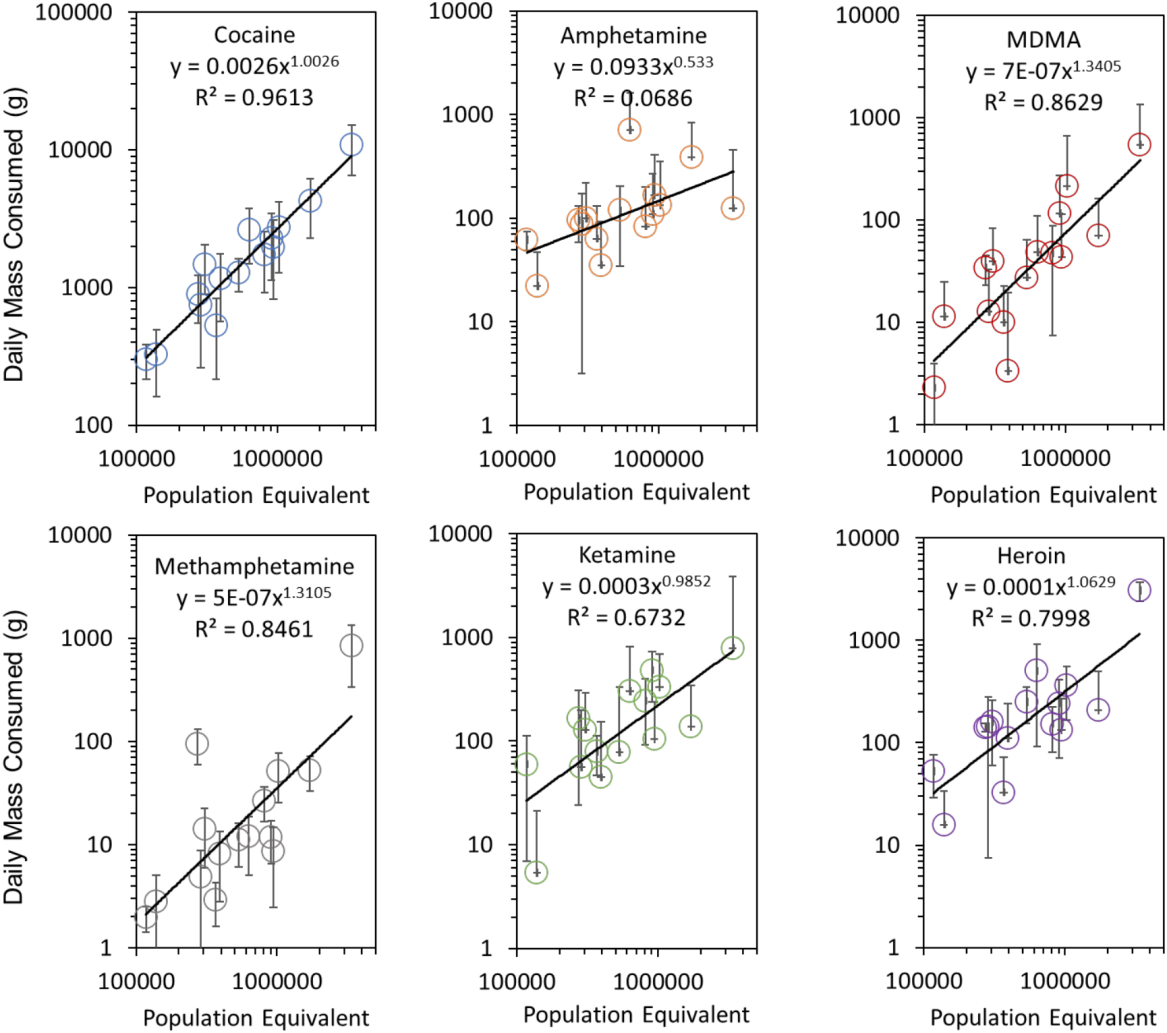
Daily mass consumed (g/day) of the six substances investigated in the 15 wastewater treatment plants (WWTPs), relative to their population equivalent.

There was no discernible positive or negative correlation between population‐normalised daily consumption in mg/1000 people/day and PE across sites (Figure S12). A direct comparison between the different WWTPs (Figure 6) revealed that the largest daily consumption by mass of substance (g/day) was in site F for five out of six substances (except amphetamine, which was largest within the site B catchment). Importantly, and as can be seen in Figure 7, the mass consumed overall could be correlated with the PE served, particularly for (in decreasing order): cocaine, MDMA, methamphetamine and heroin. Such high correlations for these substances with PEs are encouraging to enable averaging and potential generalisation to other urban sites within this PE range in the future, especially given the scale of temporal data generated here. This could have implications beyond public health, towards the early identification of site‐specific trends that could require more proactive community interventions where any evidence of inconsistent or indeed changing behaviour exists. For example, while such correlations were high in general for methamphetamine, two obvious outlier sites existed, which indicated such examples of known catchment‐specific consumption behaviour patterns, and which require deeper assessment to understand the reliability of generalisation to consumption estimations to national level. Moderate and poor correlations with PEs were observed for ketamine and amphetamine, respectively, indicating unquantifiable uncertainties in the WBE process, such as catchment‐specific behaviour. The total cumulative mass consumed for all 15 sites monitored for cocaine, heroin, ketamine, amphetamine, MDMA and methamphetamine were 12 065 ± 105 (*n* = 1729), 1698 ± 29 (*n* = 554), 1098 ± 11 (*n* = 1729), 840 ± 22 (*n* = 1729), 445 ± 18 (*n* = 1729) and 417 ± 10 (*n* = 1729) kg/year, respectively. In the case of amphetamine and ketamine, these were a lot higher than the prescribed amounts, with averages of approximately 146 kg/year of amphetamine and approximately 4.38 kg/year of ketamine prescribed in 2022 in all England (Figure S6) (approx. 30.5 and 0.92 kg/year for 20.9% of the population in this study, for amphetamine and ketamine, respectively), therefore suggesting the additional illicit use of these drugs. Amphetamine is also a metabolite of other substances, such as methamphetamine, selegiline, femprofazone, benzphetamine and clobenzorex, which may explain the higher quantities observed in WBE [66, 67, 68, 69, 70].

### Limitations and considerations

While the estimation of community drug consumption through analysis of influent wastewater has demonstrated a positive correspondence with data from epidemiological surveys, this approach carries its own limitations and uncertainties [35, 71, 72]. These mainly relate to sampling, chemical analysis and the variables used in back‐calculation (e.g. catchment population size served, stability and sorption of drug targets, exfiltration, use of averaged metabolism metrics, etc.). One obvious source of uncertainty is the reliability of the use of PE versus census data, as recently discussed by Jagadeesan *et al*. [73]. However, for a multi‐site programme, one of the main issues lies in the ability to extrapolate data to estimate national‐scale consumption. Recent studies have focused on representation based on the range of urbanicities covered [74]. Over 9000 wastewater treatment facilities operate in the UK, representing a wide range of treatment capacities and complexities. Of these, and in England alone, the Urban Wastewater Treatment Directive lists >1400 WWTPs representing PEs of sufficient size to require targeted compliance monitoring (i.e. PE > 2000 if discharging to freshwater and PE > 10 000 if discharging to coastal/transitional waters) [75]. The LSOAs covered by the 15 WWTPs sampled for WBE were 98% urban. Those not sampled were 81% urban, and all LSOAs in England were 84% urban (Table S9). The difference in the proportions was statistically significant (*P* < 0.001). This implies that despite their coverage of a large portion of the national residential population, urban areas were over‐represented. Among the sampling sites, only site E had a comparable percentage of rural LSOAs in the catchment to England‐wide proportions (17%), followed by site G, which contained 6% LSOAs classified as rural. All other sites covered areas with more than 96% urban LSOAs. In addition to this, several of the sites were prioritised to specifically cover large population centres and as known areas with high drug misuse, which may have introduced some element of bias [76]. Therefore, despite covering a wide geographical spread, the sampling coverage was not considered representative of the whole population of England, and results should therefore not be extrapolated to national level estimates (which also agrees with EUDA guidance) [77]. That said, the correlation between sites within the population range shown in Figure 7 for several substances may act as an early indicator of the relative scale of urban consumption, in particular, but still would need to be verified locally with chemical analysis for higher assurance. Furthermore, when comparing mean estimated consumption values (mg/1000 people/day) between sites in the north and the south of England, no statistically significant differences were observed for any of the five drugs tested. Wilcoxon rank‐sum tests are shown in Table S13, confirming broadly comparable estimated consumption values across sites studied in both regions.

## CONCLUSION

For the first time, high temporal and spatial resolution wastewater analysis was conducted using 1746 samples from 15 WWTPs across England to investigate trends in community consumption of 20 illicit drugs throughout 2022. Eighteen compounds were quantifiable across all sites, with only isotonitazene and its metabolite 5‐aminoisotonitazene not detected. Cocaine had the highest mean consumption (2770 ± 829 mg/1000 people/day), followed by heroin (382 ± 248 mg/1000 people/day), ketamine (287 ± 183 mg/1000 people/day), amphetamine (272 ± 268 mg/1000 people/day), MDMA (80 ± 57 mg/1000 people/day) and methamphetamine (60 ± 99 mg/1000 people/day). These sites largely represented urban areas, with some chosen for high levels of drug misuse. Compared with EUDA/SCORE data for other European cities, several English WWTP catchments ranked highly, especially for ketamine, where 13 and seven sites exceeded all other European locations, when using all‐year data and the SCORE‐aligned sampling period, respectively. Of the 7458 drug measurements recorded, 147 (4%) were extreme outliers, mainly occurring in November and December. High temporal resolution revealed spikes in PNLs following public holidays, national celebrations and major events. Although the average drug consumption remained steady across the year, a sharp decline in cocaine use occurred in March 2022, coinciding with a major UK drug seizure by law enforcement. Strong correlations between certain compounds (e.g. BZE with ketamine and cocaethylene) suggested widespread co‐consumption. Representing approximately 21% of the UK's population, this study is one of the most temporally detailed multi‐site, single‐year assessments of illicit drug consumption conducted in a single country.

## AUTHOR CONTRIBUTIONS

**Helena Rapp‐Wright:** Conceptualization (equal); data curation (lead); formal analysis (lead); investigation (equal); methodology (equal); project administration (equal); supervision (supporting); validation (lead); visualization (equal); writing—original draft (lead); writing—review and editing (equal). **Keng Tiong Ng:** Formal analysis (equal); investigation (equal). **Derryn Grant:** Formal analysis (equal). **William Francis:** Formal analysis (equal); visualization (supporting). **Margarita White:** Formal analysis (equal). **Dimitris Evangelopoulos:** Conceptualization (equal); data curation (equal); formal analysis (equal); investigation (equal); writing—original draft (equal); writing—review and editing (supporting). **Yuxing Liu:** Formal analysis (supporting); investigation (supporting). **Konstantina Dimakopoulou:** Conceptualization (equal); data curation (equal); formal analysis (equal); investigation (equal). **Sofia Zafeiratou:** Conceptualization (equal); data curation (equal); formal analysis (equal); investigation (equal). **Dylan Wood:** Formal analysis (supporting); investigation (supporting); software (supporting); validation (supporting); writing—review and editing (supporting). **Chryshanthi Christy:** Project administration (lead). **Stav Friedman:** Project administration (equal). **Timothy W. Gant:** Funding acquisition (supporting); supervision (supporting); writing—review and editing (supporting). **Klea Katsouyanni:** Conceptualization (equal); data curation (equal); formal analysis (equal); investigation (equal); methodology (equal); supervision (lead); validation (equal). **Leon P. Barron:** Conceptualization (lead); data curation (supporting); formal analysis (equal); funding acquisition (lead); investigation (lead); methodology (lead); project administration (equal); resources (lead); software (lead); supervision (lead); validation (equal); visualization (supporting); writing—original draft (lead); writing—review and editing (equal).

## DECLARATION OF INTERESTS

The authors declare that they have no known competing financial interests or personal relationships that could have appeared to influence the work reported in this article.

## Supporting information


**Figure S1.** Graphs showing LC–MSMS peak area stability over 11 days for (a) 6‐MAM and (b) its stable isotope‐labelled internal standard, 6‐MAM‐d_6_, measured in separate pre‐spiked aliquots of wastewater matrix stored at −20°C.
**Figure S2.** Top‐25 ranking of the average daily PNL data in mg/1000 people/day from English sites A–O monitored in this study represented as: (a) blue for cocaine (as BZE); (b) dark blue for methamphetamine; (c) green for ketamine; (d) red for MDMA; (e) orange for amphetamine; and in comparison with other European catchments (in grey) in 2022 reported as part of the EUDA WBE.
**Figure S3.** Heat‐map graph showing average estimated consumption (mg/1000 people/day) across all sites for all compounds investigated, normalised by drug.
**Figure S4.** Time series rolling average plot of average estimated consumption in logarithmic scale of drugs across all WWTP catchments per day per 1000 people (in mg/1000 people/day).
**Figure S5.** Box and whisker plots of estimated consumption of drugs of all WWTP catchments per day per 1000 people (in mg/1000 people/day) for all samples analysed in this study divided by quarters of the year (Q1 = January–March, Q2 = April–June, Q3 = July–September, and Q4 = October–December).
**Figure S6.** (Left) Time series rolling average plot of mass (kg/day) prescribed nationally in England from 2018 to 2024 for (a) amphetamine and (b) ketamine.
**Figure S7.** Box and whisker plots of estimated consumption (mg/1000 people/day) data comparing consumption over bank holiday weekends with regular weekends across all sites (Fridays to Mondays, inclusive) for the six compounds investigated.
**Figure S8.** Spearman's correlation matrix using paired drug PNL data (mg/1000 people/day) for all 15 sites (A–O).
**Figure S9**. Correlation between cocaine (BZE) and ketamine estimated consumption data (mg/1000 people/day) for all sites (A–O, highlighting each site data set per draft) and showing the correlation coefficient (*R* ^2^).
**Figure S10.** Box and whisker plot of the BZE/cocaethylene PNL ratio (mg/1000 people/day) across all WWTPs tested (n = 15) during (a) the weekdays (Tuesday to Thursday) and weekends (Friday to Monday) and (b) per day of the week. The blue dot represents the average value and *** means P ≤ 0.001.
**Figure S11**. Box and whisker plots per sites of the cocaethylene/BZE PNL ratio (mg/1000 people/day) during the weekdays (Tuesday–Thursday) and weekends (Friday–Monday) across all sites (A–O).
**Figure S12.** (a) Daily cocaine consumption across sites, shown both as per capita values (left) and as total mass (right). (b) Corresponding plot of per capita daily consumption (log scale) and population equivalent (PE) for all six drugs.
**Table S1.** MRM transitions used for the direct‐injection method.
**Table S2.** MRM transitions used for the SPE (6‐MAM) method.
**Table S3.** Source parameters and dwell/loop time summary for both methods.
**Table S4.** Method performance data for 19 individual compounds for direct‐injection method in wastewater matrix, based on ICH guidelines.
**Table S5.** Method performance data for 6‐MAM for SPE method in ultrapure water, based on ICH guidelines.
**Table S6.** Octanol/water partition coefficient (logP) values for compounds investigated in this study.
**Table S7.** Correlation coefficient values interpretation.
**Table S8.** Number (%) of LSOAs and population coverage by each WWTP over the area covered by sampling sites and over all areas of England using 2021 UK Census Data.
**Table S9.** Urban typology in areas with and without sampling WWTPs and by sampling WWTP in England.
**Table S10.** Descriptive statistics of daily illicit drug PNL values for each drug across all sampled WWTPs (*n* = 15) in 2022.
**Table S11.** Associations between illicit drug PNLs in wastewater across England.
**Table S12.** Pairwise number of valid daily illicit drug PNL values.|
**Table S13.** Wilcoxon rank‐sum test results comparing average estimated consumption levels (mg/1000 people/day) between sites in the North and South of England.

## Data Availability

All sharable data are available in the manuscript or in the supplementary information document.
