# Supplementary material for: High temporal resolution monitoring of illicit drug consumption across England via wastewater analysis
Source: Addiction. 2026 Apr 26;121(8):2043–59. doi: 10.1111/add.70398 (PMC13357825; doi:10.1111/add.70398)
Supplement: Supplementary file 1 — Figure S1. Graphs showing LC–MSMS peak area stability over 11 days for (a) 6‐MAM and (b) its stable isotope‐labelled internal standard, 6‐MAM‐d6, measured in separate pre‐spiked aliquots of wastewater matrix stored at −20°C. Figure S2. Top‐25 ranking of the average daily PNL data in mg/1000 people/day from English sites A–O monitored in this study represented as: (a) blue for cocaine (as BZE); (b) dark blue for methamphetamine; (c) green for ketamine; (d) red for MDMA; (e) orange for amphetamine; and in comparison with other European catchments (in grey) in 2022 reported as part of the EUDA WBE. Figure S3. Heat‐map graph showing average estimated consumption (mg/1000 people/day) across all sites for all compounds investigated, normalised by drug. Figure S4. Time series rolling average plot of average estimated consumption in logarithmic scale of drugs across all WWTP catchments per day per 1000 people (in mg/1000 people/day). Figure S5. Box and whisker plots of estimated consumption of drugs of all WWTP catchments per day per 1000 people (in mg/1000 people/day) for all samples analysed in this study divided by quarters of the year (Q1 = January–March, Q2 = April–June, Q3 = July–September, and Q4 = October–December). Figure S6. (Left) Time series rolling average plot of mass (kg/day) prescribed nationally in England from 2018 to 2024 for (a) amphetamine and (b) ketamine. Figure S7. Box and whisker plots of estimated consumption (mg/1000 people/day) data comparing consumption over bank holiday weekends with regular weekends across all sites (Fridays to Mondays, inclusive) for the six compounds investigated. Figure S8. Spearman's correlation matrix using paired drug PNL data (mg/1000 people/day) for all 15 sites (A–O). Figure S9. Correlation between cocaine (BZE) and ketamine estimated consumption data (mg/1000 people/day) for all sites (A–O, highlighting each site data set per draft) and showing the correlation coefficient (R 2). Figure S10. Box and whisker plot of th [file ADD-121-2043-s001.docx]

**HIGH TEMPORAL RESOLUTION MONITORING OF ILLICIT DRUG CONSUMPTION ACROSS ENGLAND VIA WASTEWATER ANALYSIS**

Helena Rapp-Wright^a^, Keng Tiong Ng^a^, Derryn Grant^a,b^, William Francis^a,b^, Margarita White^a^, Dimitris Evangelopoulos^a,b^, Yixing Liu^a^, Konstantina Dimakopoulou^c^, Sofia Zafeiratou^c^, Dylan Wood^a^, Chryshanthi Christy^a^, Stav Friedman^a^, Timothy W. Gant,^d^ Klea Katsouyanni^a,b,c^_,_ and Leon P. Barron^a, b^*

^a^MRC Centre for Environment and Health, Environmental Research Group, School of Public Health, Imperial College London, Wood Lane, London W12 0BZ, United Kingdom

^b^NIHR HPRU in Environmental Exposures and Health, Imperial College London, United Kingdom

^c^Department of Hygiene, Epidemiology and Medical Statistics, Medical School, National and Kapodistrian University of Athens, Athens, Greece

^d^UK Health Security Agency, Harwell Science Campus, Femi Avenue, Harwell, Didcot OX11 0GD, UK

*Corresponding author E-mail address: [leon.barron@imperial.ac.uk](mailto:h.rapp-wright@imperial.ac.uk)

Instrumental analysis procedures

*Direct-injection LC-MS/MS gradient elution profile*

Optimised gradient elution conditions were as follows: 10 % mobile phase B for 0.2 min; a linear ramp from 10 to 60 % from 0.2 to 3.0 min; a step gradient from 60 to 100 % at 3.1 min; held at 100 % B to 4.0 min; and decreased from 100 to 10 % at 4.01 min before re-equilibration time until 6.5 min. Between runs, a 30 s period was also necessary for needle washing (acetonitrile) and autosampler cycling for the next sample.

*LC-MS/MS gradient elution profile for 6-MAM after SPE*

Optimised gradient elution conditions were as follows: 20 % mobile phase B for 0.2 min; a linear ramp from 20 to 60 % from 0.2 to 0.8 min; a step gradient from 60 to 100 % at 1.01 min; held at 100 % B to 1.7 min; and decreased from 100 to 20 % at 1.71 min before re-equilibration time until 3 min. Between runs, a 30 s period was also necessary for needle washing (acetonitrile) and autosampler cycling for the next sample.

**Table S1.** MRM transitions used for the direct-injection method.

| **Compounds** | **Precursor (m/z)** | **Transition (m/z)** | **Polarity** | **Pause Time (ms)** | **Dwell Time (ms)** | **Q1 Pre Bias** | **Collision Energy (V)** | **Q3 Pre Bias** | **Retention Time Window Range (min)** | |
| --- | --- | --- | --- | --- | --- | --- | --- | --- | --- | --- |
|  |  |  |  |  |  | **(V)** |  | **(V)** |  |  |
| 5-aminoisotonitazene | 381.1 | 100.1 | + | 2.0 | 10.0 | -19 | -22 | -10 | 1.475 | 2.275 |
|  |  | 72.2 | + | 2.0 | 10.0 | -19 | -48 | -13 |  |  |
| 6-acetylcodeine | 342.0 | 225.1 | + | 2.0 | 10.0 | -18 | -28 | -14 | 1.932 | 2.732 |
|  |  | 301.2 | + | 2.0 | 10.0 | -18 | -12 | -20 |  |  |
| Amphetamine | 136.0 | 90.85 | + | 2.0 | 2.0 | -20 | -15 | -20 | 0.898 | 1.698 |
|  |  | 118.9 | + | 2.0 | 2.0 | -19 | -13 | -12 |  |  |
|  |  | 65.0 | + | 2.0 | 2.0 | -18 | -37 | -26 |  |  |
| Amphetamine-d_6_ | 142.2 | 93.2 | + | 2.0 | 4.0 | -17 | -19 | -17 | 0.898 | 1.698 |
|  |  | 125.2 | + | 2.0 | 4.0 | -16 | -14 | -21 |  |  |
| Benzocaine | 166.0 | 138.3 | + | 2.0 | 1.0 | -10 | -17 | -25 | 2.265 | 3.065 |
|  |  | 77.2 | + | 2.0 | 1.0 | -12 | -27 | -30 |  |  |
|  |  | 94.1 | + | 2.0 | 1.0 | -10 | -21 | -17 |  |  |
| BZE | 289.9 | 168.0 | + | 2.0 | 1.0 | -20 | -20 | -20 | 1.619 | 2.419 |
|  |  | 76.9 | + | 2.0 | 1.0 | -17 | -54 | -15 |  |  |
|  |  | 104.95 | + | 2.0 | 1.0 | -18 | -31 | -21 |  |  |
| BZE-d_3_ | 293.1 | 177.1 | + | 2.0 | 9.0 | -30 | -20 | -18 | 1.619 | 2.419 |
| Cocaethylene | 318.2 | 196.0 | + | 2.0 | 10.0 | -20 | -20 | -20 | 2.323 | 3.123 |
|  |  | 82.1 | + | 2.0 | 10.0 | -19 | -30 | -20 |  |  |
|  |  | 150.2 | + | 2.0 | 10.0 | -12 | -26 | -15 |  |  |
| Cocaine | 304.1 | 181.9 | + | 2.0 | 1.0 | -20 | -20 | -20 | 2.008 | 2.808 |
|  |  | 76.9 | + | 2.0 | 1.0 | -20 | -50 | -20 |  |  |
|  |  | 81.9 | + | 2.0 | 1.0 | -20 | -30 | -20 |  |  |
| Cocaine-d_3_ | 307.1 | 185.2 | + | 2.0 | 3.0 | -22 | -21 | -19 | 2.008 | 2.808 |
|  |  | 77.1 | + | 2.0 | 3.0 | -15 | -54 | -14 |  |  |
| EDDP | 279.0 | 235.1 | + | 2.0 | 10.0 | -14 | -32 | -15 | 2.783 | 3.583 |
| Isotonitazene | 411.1 | 100.1 | + | 2.0 | 10.0 | -12 | -25 | -10 | 2.668 | 3.468 |
|  |  | 370.1 | + | 2.0 | 10.0 | -15 | -9 | -18 |  |  |
| Ketamine | 238.1 | 125.0 | + | 2.0 | 3.0 | -12 | -26 | -26 | 1.627 | 2.427 |
|  |  | 207.0 | + | 2.0 | 3.0 | -12 | -15 | -15 |  |  |
| Ketamine-d_4_ | 242.1 | 129.2 | + | 2.0 | 3.0 | -25 | -28 | -26 | 1.627 | 2.427 |
|  |  | 211.2 | + | 2.0 | 3.0 | -28 | -15 | -10 |  |  |
| Levamisole | 205.1 | 178.0 | + | 2.0 | 1.0 | -20 | -24 | -21 | 1.27 | 2.07 |
|  |  | 91.0 | + | 2.0 | 1.0 | -14 | -37 | -16 |  |  |
|  |  | 123.2 | + | 2.0 | 1.0 | -14 | -29 | -12 |  |  |
| Lidocaine | 235.0 | 85.9 | + | 2.0 | 1.0 | -20 | -20 | -20 | 1.255 | 2.055 |
|  |  | 57.9 | + | 2.0 | 1.0 | -13 | -35 | -20 |  |  |
|  |  | 29.9 | + | 2.0 | 1.0 | -14 | -51 | -11 |  |  |
| Lidocaine-d_10_ | 245.2 | 96.3 | + | 2.0 | 9.0 | -29 | -22 | -17 | 1.255 | 2.055 |
| MDMA | 194.1 | 105.1 | + | 2.0 | 1.0 | -10 | -25 | -22 | 1.248 | 2.048 |
|  |  | 163.0 | + | 2.0 | 1.0 | -10 | -13 | -12 |  |  |
| MDMA-d_5_ | 199.1 | 165.2 | + | 2.0 | 3.0 | -22 | -14 | -29 | 1.248 | 2.048 |
|  |  | 107.2 | + | 2.0 | 3.0 | -10 | -25 | -11 |  |  |
| Methadone | 310.3 | 265.0 | + | 2.0 | 10.0 | -20 | -15 | -20 | 3.008 | 3.808 |
|  |  | 105.0 | + | 2.0 | 10.0 | -20 | -35 | -20 |  |  |
|  |  | 57.2 | + | 2.0 | 10.0 | -11 | -24 | -23 |  |  |
| Methamphetamine | 150.0 | 91.0 | + | 2.0 | 2.0 | -10 | -19 | -18 | 1.092 | 1.892 |
|  |  | 65.0 | + | 2.0 | 2.0 | -22 | -40 | -20 |  |  |
|  |  | 118.9 | + | 2.0 | 2.0 | -20 | -14 | -20 |  |  |
| Methamphetamine-d_5_ | 155.15 | 92.1 | + | 2.0 | 2.0 | -16 | -22 | -16 | 1.092 | 1.892 |
|  |  | 114.2 | + | 2.0 | 2.0 | -16 | -12 | -21 |  |  |
|  |  | 60.1 | + | 2.0 | 2.0 | -18 | -15 | -25 |  |  |
| Morphine | 286.1 | 153.1 | + | 2.0 | 5.0 | -14 | -45 | -29 | 0.645 | 1.445 |
|  |  | 165.1 | + | 2.0 | 5.0 | -14 | -44 | -12 |  |  |
| Morphine-d_3_ | 289.0 | 152.1 | + | 2.0 | 5.0 | -14 | -55 | -30 | 0.645 | 1.445 |
|  |  | 201.1 | + | 2.0 | 5.0 | -14 | -27 | -21 |  |  |
| Norketamine | 224.9 | 208.2 | + | 2.0 | 10.0 | -27 | -12 | -22 | 1.516 | 2.316 |
|  |  | 126.2 | + | 2.0 | 10.0 | -11 | -25 | -24 |  |  |
|  |  | 125.1 | + | 2.0 | 10.0 | -11 | -24 | -12 |  |  |
| Phenacetin | 180.1 | 110.0 | + | 2.0 | 1.0 | -20 | -20 | -20 | 2.077 | 2.877 |
|  |  | 64.9 | + | 2.0 | 1.0 | -20 | -35 | -20 |  |  |
|  |  | 92.9 | + | 2.0 | 1.0 | -20 | -30 | 20 |  |  |
| Procaine | 237.2 | 119.9 | + | 2.0 | 2.0 | -20 | -25 | -20 | 1.055 | 1.855 |
|  |  | 163.8 | + | 2.0 | 2.0 | -20 | -16 | -20 |  |  |

**Table S2.** MRM transitions used for the SPE (6-MAM) method.

| **Compounds** | **Precursor (m/z)** | **Transition (m/z)** | **Polarity** | **Pause Time (ms)** | **Dwell Time (ms)** | **Q1 Pre Bias** | **Collision Energy (V)** | **Q3 Pre Bias** | **Retention Time Window Range (min)** | |
| --- | --- | --- | --- | --- | --- | --- | --- | --- | --- | --- |
|  |  |  |  |  |  | **(V)** |  | **(V)** |  |  |
| 6-MAM | 328.0 | 164.9 | + | 2.0 | 1.0 | -20 | -38 | -17 | 0.535 | 1.535 |
|  |  | 210.8 | + | 2.0 | 1.0 | -20 | -26 | -22 |  |  |
|  |  | 192.8 | + | 2.0 | 1.0 | -20 | -28 | -20 |  |  |
| 6-MAM-d_6_ | 334.0 | 165.0 | + | 2.0 | 3.0 | -17 | -40 | -30 | 0.535 | 1.535 |
|  |  | 210.9 | + | 2.0 | 3.0 | -10 | -28 | -15 |  |  |

**Table S3.** Source parameters and dwell/loop time summary for both methods.

| **ESI Interface Conditions** | | |
| --- | --- | --- |
| Nebulising gas flow, L/min | 2 | |
| Heating gas flow, L/min | 15 | |
| Interface temperature, °C | 400 | |
| DL temperature, °C | 250 | |
| Heat block temperature, °C | 400 | |
| Drying gas flow, L/min | 3 | |
|  |  | |
| **Dwell Time/Loop Time** | | |
|  | **Direct-injection** | **SPE (6-MAM)** |
| Maximum event | 20 | 2 |
| Maximum dwell time, ms | 10 | 3 |
| Minimum dwell time, ms | 1 | 1 |
| Maximum loop time, s | 0.080 | 0.019 |

Method performance

Both methods were assessed using the International Council for Harmonisation of Technical Requirements for Pharmaceuticals for Human Use (ICH) guidelines (1). Parameters of linearity, range, limits of detection and quantification (LOD and LOQ, respectively), accuracy, precision and matrix effects were investigated using pooled samples as matrix for the direct-injection method and ultrapure water for the SPE method. Subtraction of average peak areas in the unspiked sample (n = 3) was performed for analytes already present in the matrix. Matrix effects (ME) were determined at two concentrations (n = 6) and was performed by comparing the peak area of the compound in wastewater (subtracted from the blank if present in sample) to the peak area of the compound in a standard prepared in wastewater and ultrapure water, respectively and expressed as percentage. Recoveries were also examined for 6-MAM when using SPE for sample preparation at 10 ng·L^-1^ (n=6).

**Table S4.** Method performance data of 19 individual compounds for direct-injection method in wastewater matrix based on ICH guidelines.

| **Compound** | **Range**  **(ng∙L^-1^)** | **Linearity (R^2^)**  **n≥5** | **LOD**  **(ng∙L^-1^)^b^** | **LOQ**  **(ng∙L^-1^)^b^** | **Inaccuracy (%), n=6** | | | **Imprecision (%), n=6** | | | **ME (%), n=6** | |
| --- | --- | --- | --- | --- | --- | --- | --- | --- | --- | --- | --- | --- |
|  |  |  |  |  | **L** | **M** | **H** | **L** | **M** | **H** | **L** | **H** |
| 5-aminoisotonitazene^a^ | 10-7,500 | 0.9981 | 2 | 6 | 17 | 2 | -3 | 24 | 18 | 7 | -19 | -26 |
| 6-acetylcodeine^a^ | 5-7,500 | 0.9988 | 1 | 4 | 1 | 1 | 0 | 3 | 1 | 4 | -29 | -41 |
| Amphetamine* | 50-20,000 | 0.9811 | 10 | 29 | 0 | 2 | 2 | 21 | 16 | 10 | 0 | 1 |
| Benzocaine | 10-20,000 | 0.9904 | 2 | 7 | -1 | 0 | 1 | 25 | 16 | 8 | 37 | -8 |
| BZE* | 10-20,000 | 0.9912 | 2 | 7 | -12 | -5 | 2 | 27 | 14 | 4 | 401 | 36 |
| Cocaethylene^a^ | 10-7,500 | 0.9876 | 2 | 7 | 12 | 4 | -1 | 14 | 17 | 3 | -61 | -63 |
| Cocaine* | 5-20,000 | 0.9944 | 2 | 5 | 2 | 1 | 1 | 5 | 15 | 9 | 28 | -52 |
| EDDP^a^ | 5-7,500 | 0.9936 | 1 | 3 | -1 | 1 | 0 | 1 | 1 | 3 | 33 | 27 |
| Isotonitazene^a^ | 10-20,000 | 0.9981 | 3 | 7 | -3 | 0 | 2 | 30 | 12 | 2 | 49 | 45 |
| Ketamine* | 5-20,000 | 0.9946 | 2 | 5 | 1 | 1 | -1 | 12 | 10 | 10 | 2 | 17 |
| Levamisole | 10-20,000 | 0.9877 | 3 | 9 | -2 | -1 | 0 | 62 | 18 | 14 | 84 | -7 |
| Lidocaine* | 10-20,000 | 0.9886 | 2 | 6 | -2 | 1 | 1 | 13 | 6 | 8 | 17 | 10 |
| MDMA* | 5-20,000 | 0.9976 | 2 | 5 | -3 | 1 | 1 | 29 | 12 | 11 | -2 | -18 |
| Methadone^a^ | 10-7,500 | 0.9864 | 2 | 7 | 10 | 2 | -1 | 9 | 7 | 2 | 61 | 22 |
| Methamphetamine* | 5-20,000 | 0.9990 | 1 | 4 | -2 | 1 | 0 | 4 | 4 | 7 | 3 | -6 |
| Morphine* | 10-20,000 | 0.9835 | 2 | 7 | -4 | -1 | -1 | 26 | 9 | 10 | 7 | -38 |
| Norketamine^a^ | 25-7,500 | 0.9841 | 4 | 12 | 10 | 3 | 0 | 6 | 26 | 6 | -5 | -15 |
| Phenacetin | 5-20,000 | 0.9990 | 1 | 4 | -1 | 0 | 0 | 25 | 7 | 4 | -20 | -9 |
| Procaine | 5-20,000 | 0.9967 | 1 | 4 | -1 | 1 | 1 | 11 | 4 | 5 | -24 | -31 |

^a^Performed at 125, 1250 and 3,000 ng∙L^-1^. ME performed at 300 and 1,500 ng∙L^-1^. Rest of compounds performed at 250, 750 and 2,500 ng∙L^-1^. ME performed at 100 and 1,000 ng∙L^-1^.

^b^Based on the 3.3 (LOD) and 10 times (LOQ) standard error of the calibration curve intercept.

*Using the peak area ratio method. SIL-IS concentration = 500 ng∙L^-1^.

**Table S5.** Method performance data of 6-MAM for SPE method in ultrapure water based on ICH guidelines.

| **Compound** | **Range**  **(ng∙L^-1^)** | **Linearity (R^2^)**  **n≥5** | **LOD**  **(ng∙L^-1^)^a^** | **LOQ** **(ng∙L^-1^)^a^** | **Inaccuracy (%), n=6** | | | **Imprecision (%), n=6** | | | **Relative recovery (%), n=6** |
| --- | --- | --- | --- | --- | --- | --- | --- | --- | --- | --- | --- |
|  |  |  |  |  | **8 ng∙L^-1^** | **25 ng∙L^-1^** | **35 ng∙L^-1^** | **8 ng∙L^-1^** | **25 ng∙L^-1^** | **35 ng∙L^-1^** | **10 ng∙L^-1^** |
| 6-MAM* | 5-75 | 0.9970 | 1 | 4 | -3 | 0 | 0 | 5 | 4 | 3 | 107 ± 13 |
| ^a^Based on the 3.3 (LLOD) and 10 times (LLOQ) standard error of the calibration curve intercept.  *Using the peak area ratio method. SIL-IS concentration = 10 ng∙L^-1^. | | | | | | | | | | | |

Back-calculations for estimation consumption per population

PNLs were calculated using the measured DTR concentrations following Equation S1:

***PNL (mg/1000 people/day) = PE x DTR Concentration x Flow x 10^-8^* (Equation S1)**

where: DTR concentration = the concentration of the DTR measured by LC-MS/MS in ng/L.

Flow = represents the total daily wastewater flow entering the WWTP (m^3^/day).

PE (Population Equivalent) = term used to describe the size of wastewater discharge.

To translate PNL data to the amount consumed by a population, a correction factor for each drug according to Equation S2.

***Narcotic Estimated Consumption (mg/1000 people/day) = PNL x Correction Factor* (Equation S2)**

**Table S6.** Octanol/water partition coefficient (logP) values for compounds investigated in this study. Data sourced from PubChem unless specified otherwise (<https://pubchem.ncbi.nlm.nih.gov/>).

| **Compound** | **LogP** | |
| --- | --- | --- |
|  | Experimental | Calculated |
| 5-aminoisotonitazene | - | 4.2^a^ |
| 6-acetylcodeine | - | 1.7^a^ |
| 6-MAM | - | 1.6 |
| Amphetamine | 1.8 | 1.8 |
| Benzocaine | - | 1.86^b^ |
| BZE | -1.3 | 2.3 |
| Cocaethylene | - | 2.8 |
| Cocaine | 2.3 | 2.3 |
| EDDP | - | 5 |
| Isotonitazene | - | 4.7^a^ |
| Ketamine | 3.1 | 3.0 |
| Levamisole | 2.2^a^ | 2.3^b^ |
| Lidocaine | - | 2.1^c^ |
| MDMA | - | 2.1 |
| Methadone | 3.9 | 3.9 |
| Methamphetamine | 2.1 | 2.2 |
| Morphine | -0.1 | 0.9 |
| Norketamine | - | 2.4 |
| Phenacetin | - | 1.58^c^ |
| Procaine | - | 1.92^c^ |
| ^a^Baker DR, Kasprzyk-Hordern B (2011) *Journal of Chromatography A* (2).  ^b^HMDB Human Metabolome Database (HMDB).  ^b^Hazardous Substances Data Bank (HSDB). | | |

Statistical and data analysis

**Table S7.** Correlation coefficient values interpretation.

| **Correlation coefficient value (R^2^)** | **Strength of correlation** |
| --- | --- |
| 0.80 – 1.00 | Very strong |
| 0.60 – 0.799 | Strong |
| 0.40 – 0.599 | Medium |
| 0.20 – 0.399 | Weak |
| 0.00 – 0.199 | Very weak |

**
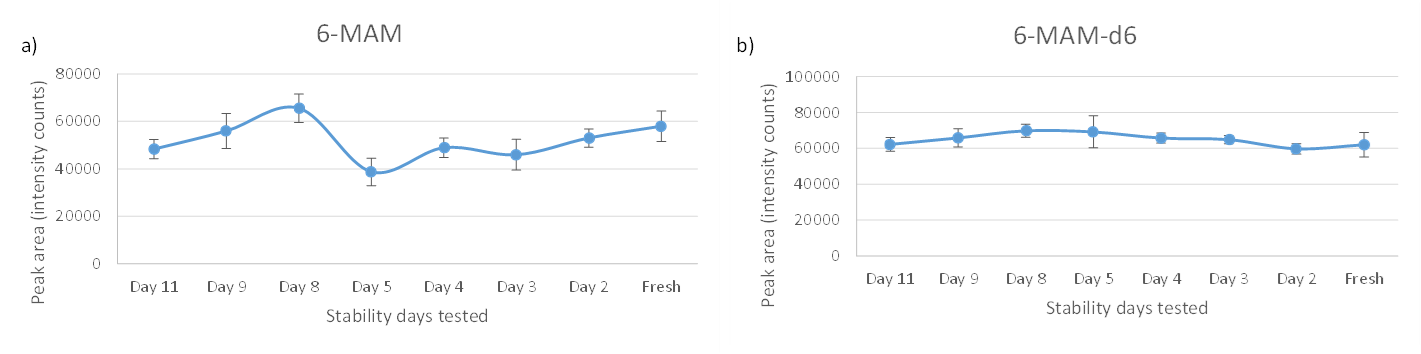
**

**Figure S1.** Graphs showing LC-MSMS peak area stability over 11 days for 6-MAM (a) and its stable isotope-labelled internal standard, 6-MAM-d_6_ (b) measured in separate pre-spiked aliquots of wastewater matrix stored at –20 ^o^C. Dots represent the average of the measured peak area for n=3 injections of the same aliquot and whiskers represent the standard deviation.


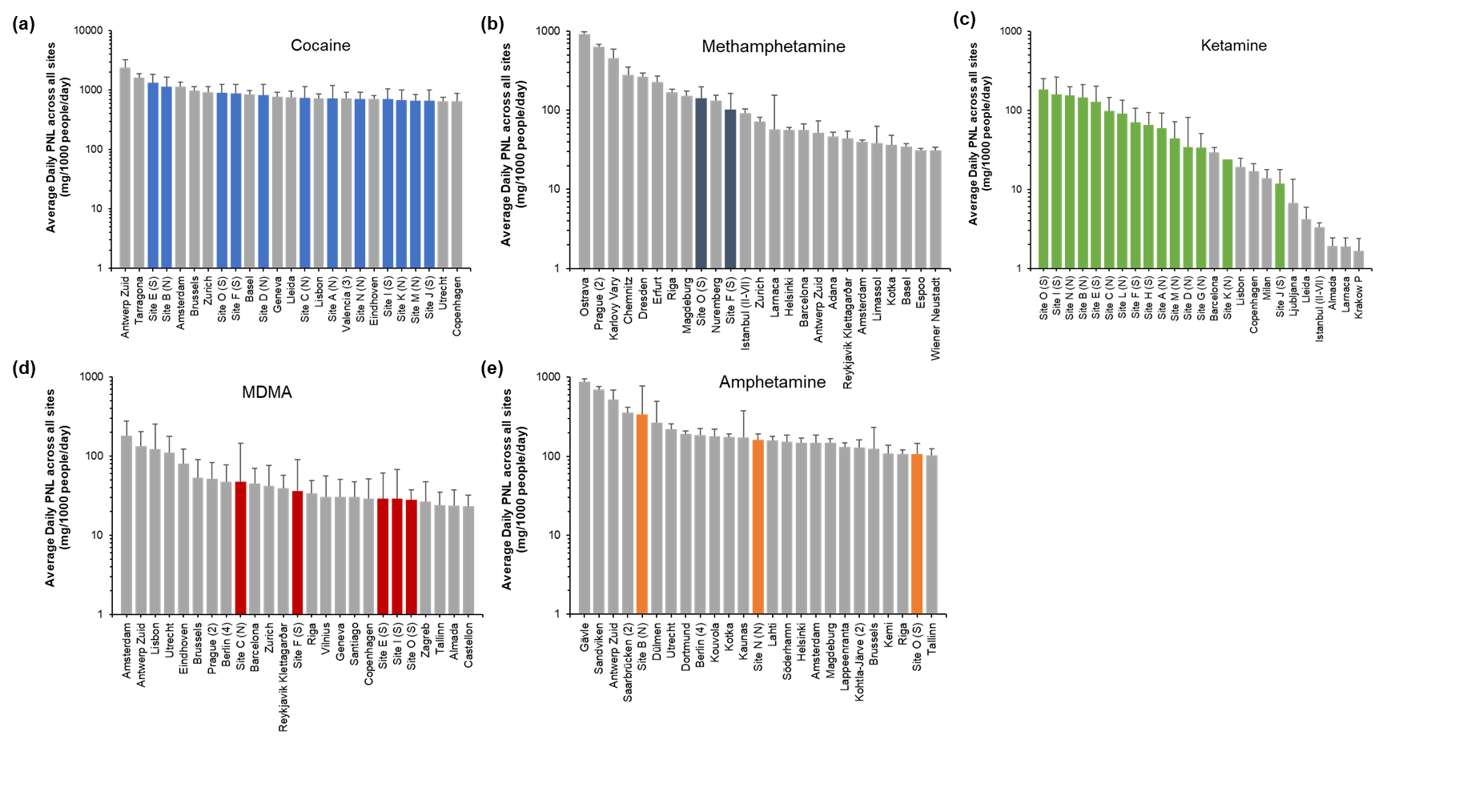


**Figure S2.** Top-25 ranking of the average daily PNL data in mg/1000 people/day from English sites A-O monitored in this study represented as (a) blue for cocaine (as BZE), (b) dark blue for methamphetamine, (c) green for ketamine, (d) red for MDMA and (e) orange for amphetamine and in comparison to other European catchments (in grey) in 2022 reported as part of the EUDA WBE. Cities with multiple WWTPs have the number of WWTPs monitored listed in parenthesis. Note: no EUDA data reported for 6-MAM in 2022 for comparison. Bracketed letters indicate regional classification, where (N) corresponds to the North of England and (S) to the South of England.


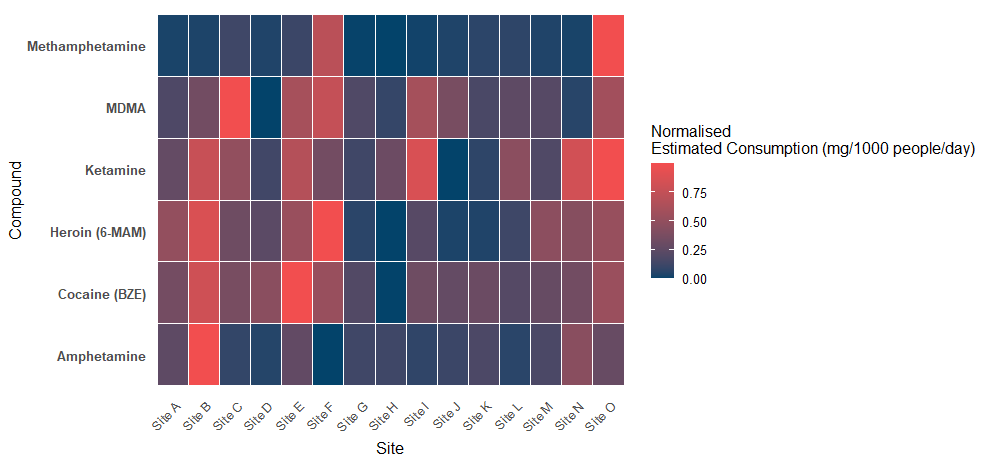


**Figure S3.** Heatmap graph showing average estimated consumption (mg/1000 people/day) across all sites for all compounds investigated, normalised by drug; where darker the colour means a higher estimated consumption determined, with blue indicating the lowest and red indicating the highest values observed for each compound.


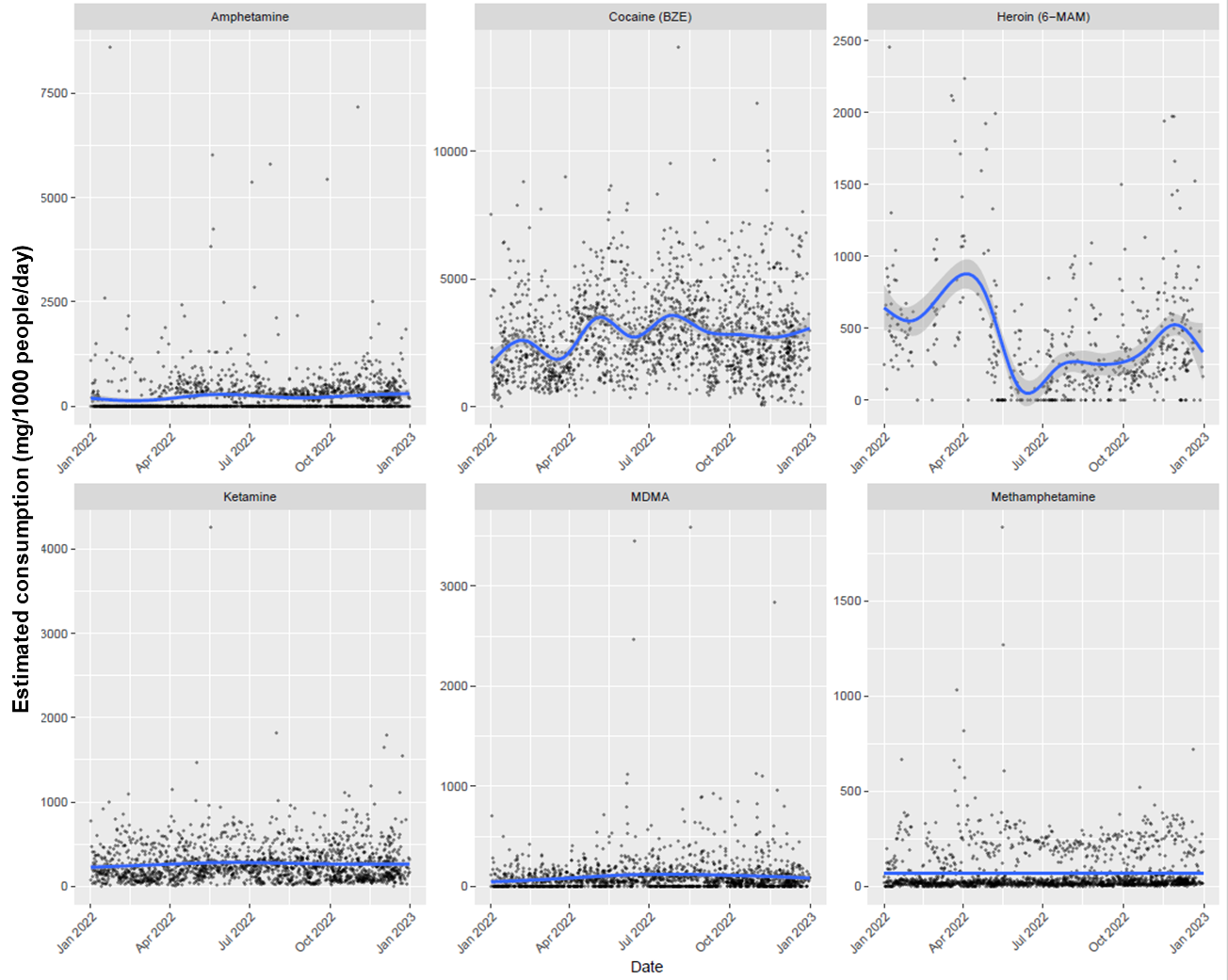

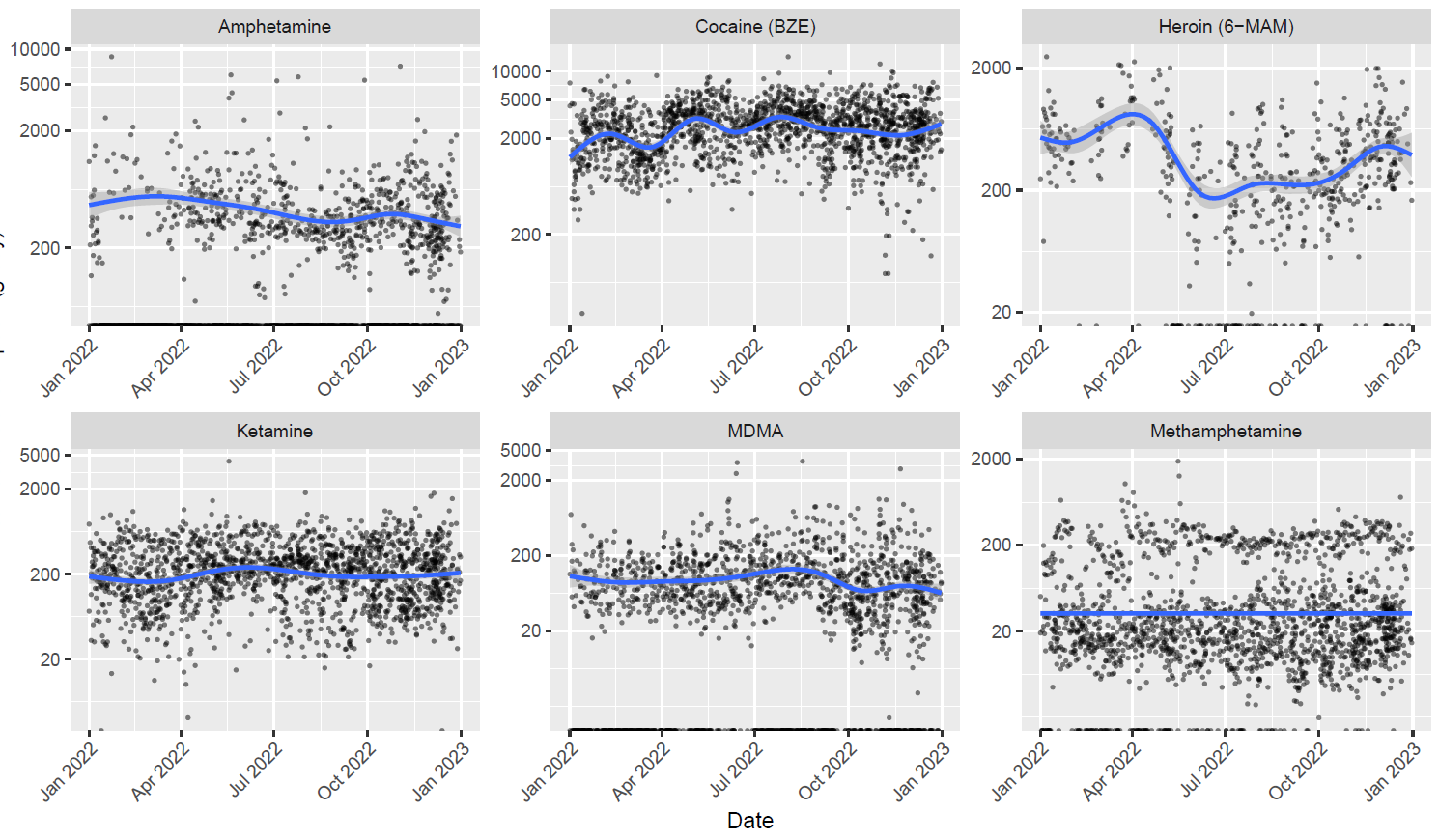


**Figure S4.** Time series rolling average plot of average estimated consumption in logarithmic scale of drugs across all WWTP catchments per day per 1000 people (in mg/1000 people/day). Confidence interval bands of 95 % are shown in grey.


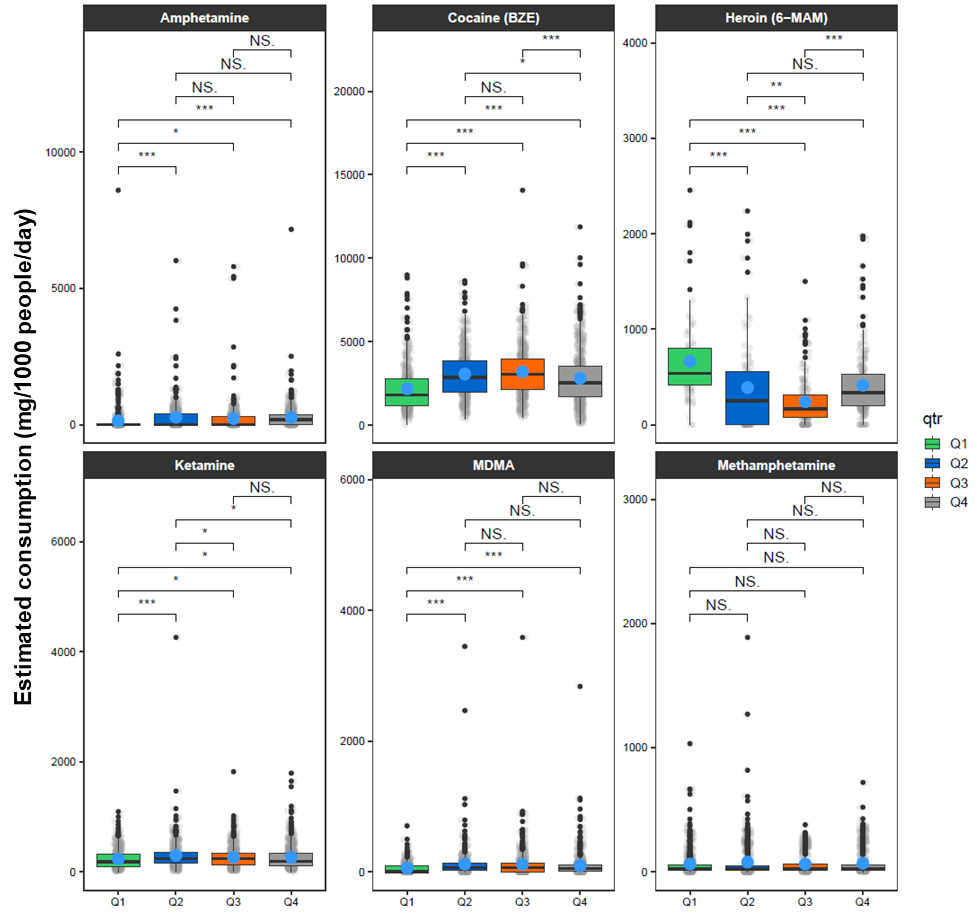


**Figure S5.** Box and whisker plots of estimated consumption of drugs of all WWTP catchments per day per 1000 people (in mg/1000 people/day) for all samples analysed in this study divided by quarters of the year (Q1 = January-March, Q2 = April-June, Q3 = July-September, and Q4 = October-December); where NS. means not significant (P >0.05), * means P ≤0.05, ** means P ≤0.01 and *** means P ≤0.001, and blue dots represent the average.

**
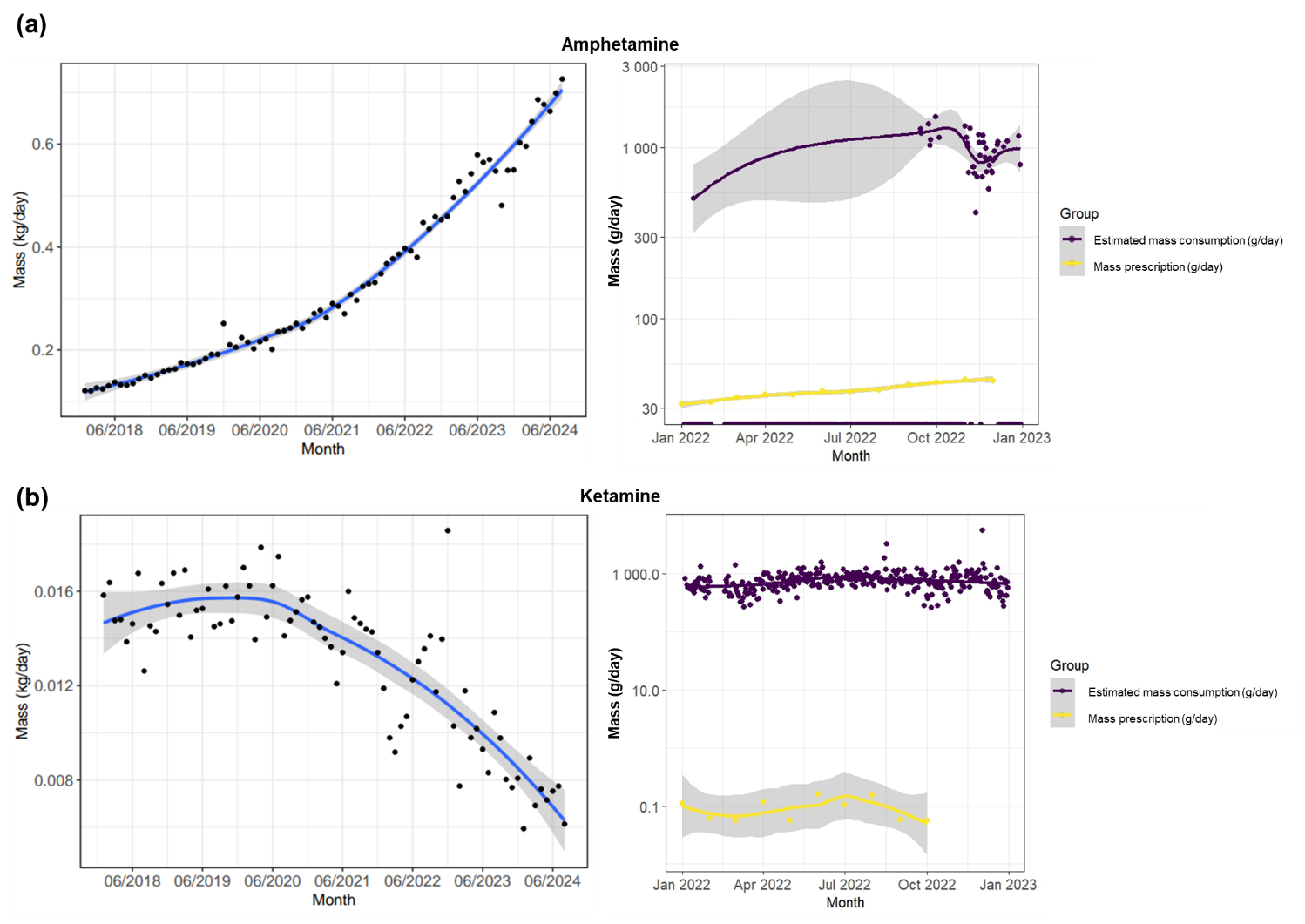
**

**Figure S6.** (Left) Time series rolling average plot of mass (kg/day) prescribed nationally in England from 2018 to 2024 for (a) amphetamine and (b) ketamine. (Right) Time series rolling average plot of estimated daily mass consumption (g/day) and mass prescribed (g/day) in Site F (i.e., calculated using Site F Lower Layer Super Output Areas (LSOAs)) for (a) amphetamine and (b) ketamine using logarithmic scale. Confidence interval bands of 95 % are shown in grey.


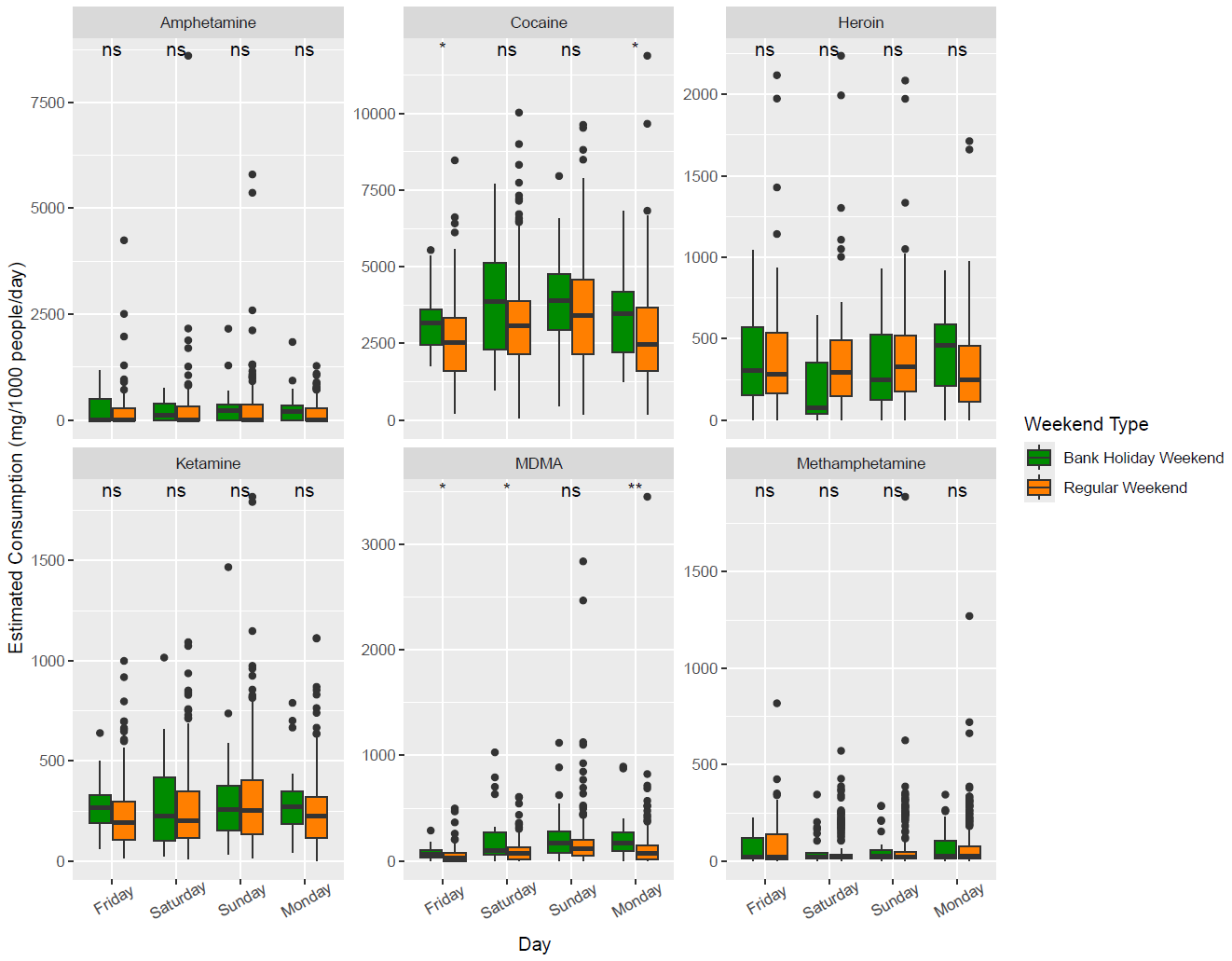


**Figure S7.** Box and whisker plots of estimated consumption (mg/1000 people/day) data comparing consumption over bank holiday weekends to regular weekends across all sites (Fridays to Mondays, inclusive) for the six compounds investigated; where ns means not significant (P >0.05), * means P ≤0.05, and ** means P ≤0.01.


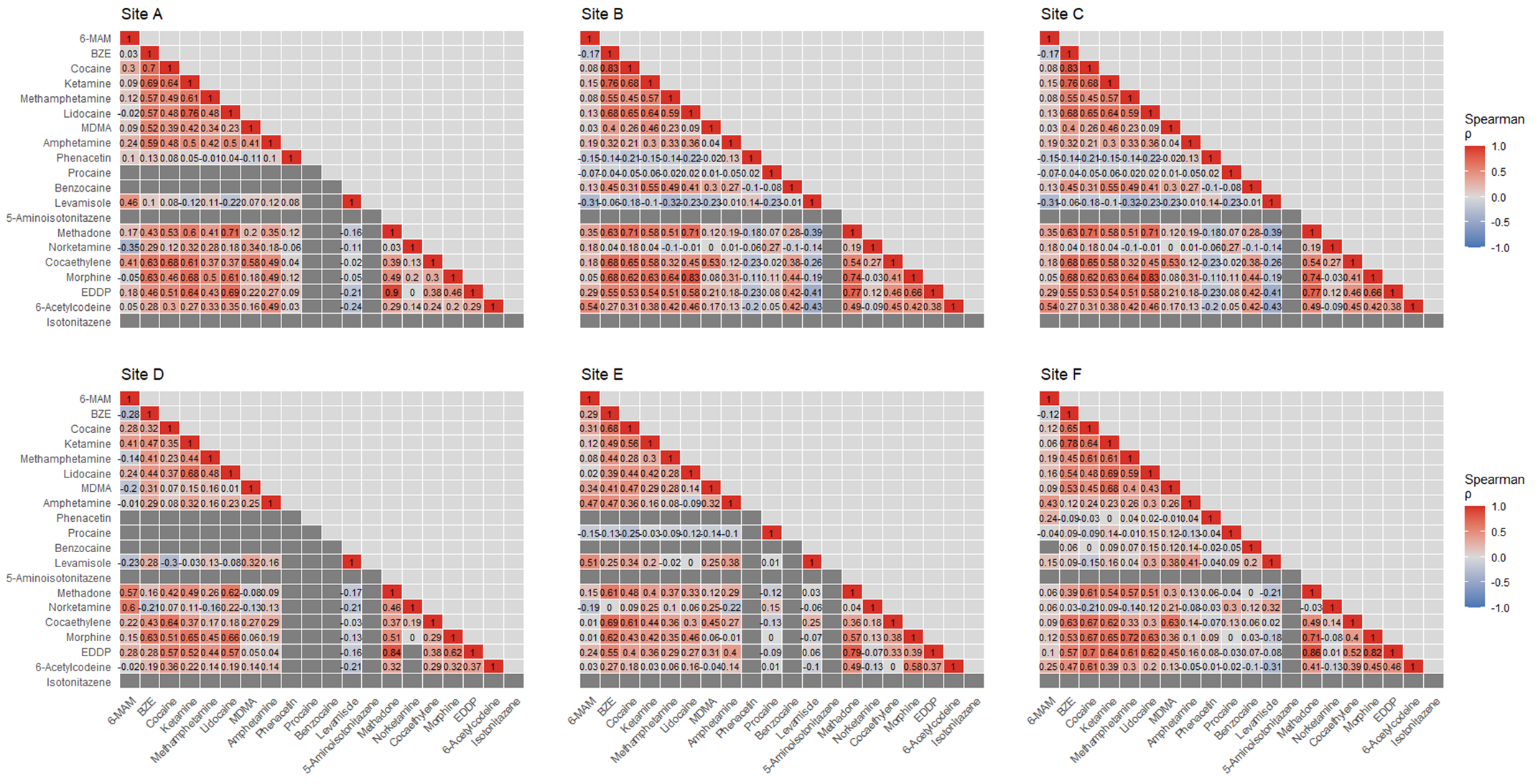
**Figure S8.** Spearman’s correlation matrix using paired drug PNL data (mg/1000 people/day) for all 15 sites (i.e., A to O). Colour intensity reflects the strength and direction of the Spearman correlation coefficient (ρ), with numerical values shown in each cell. Dark grey cells indicate instances where correlations were not possible.

**Figure S8. continued**

**
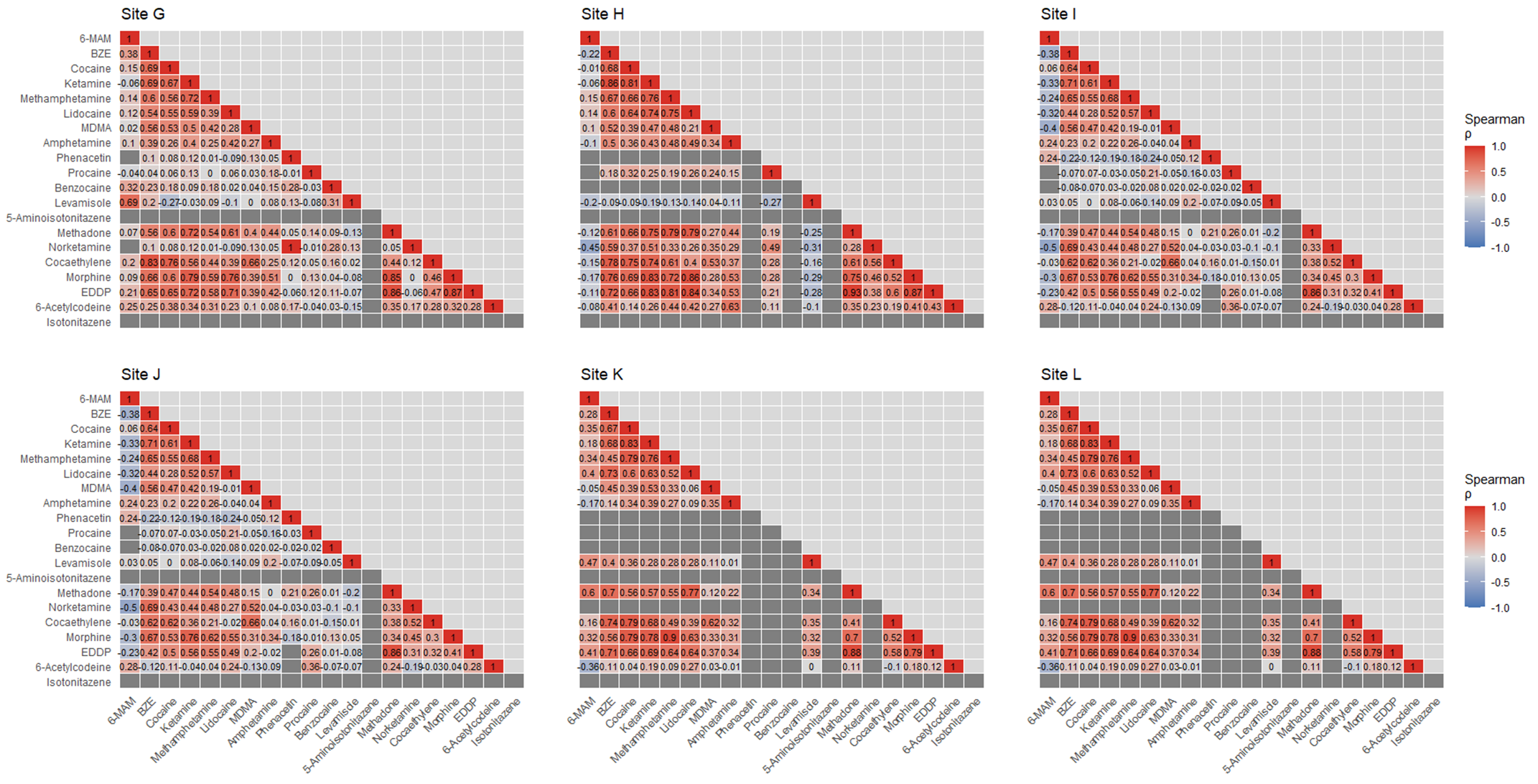
**

**Figure S8. continued**

**
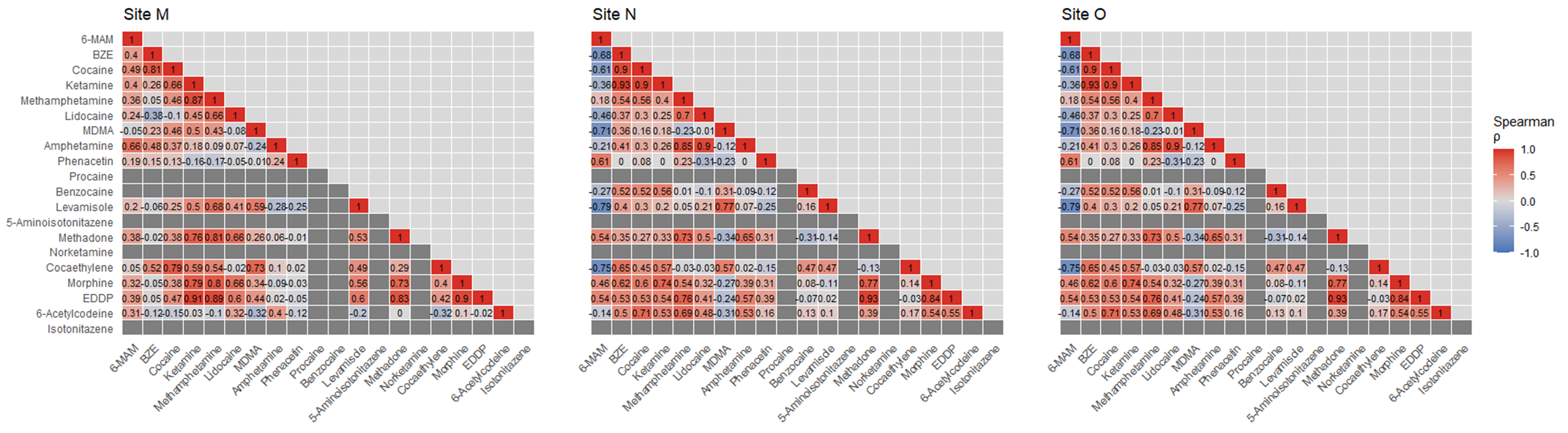
**

**
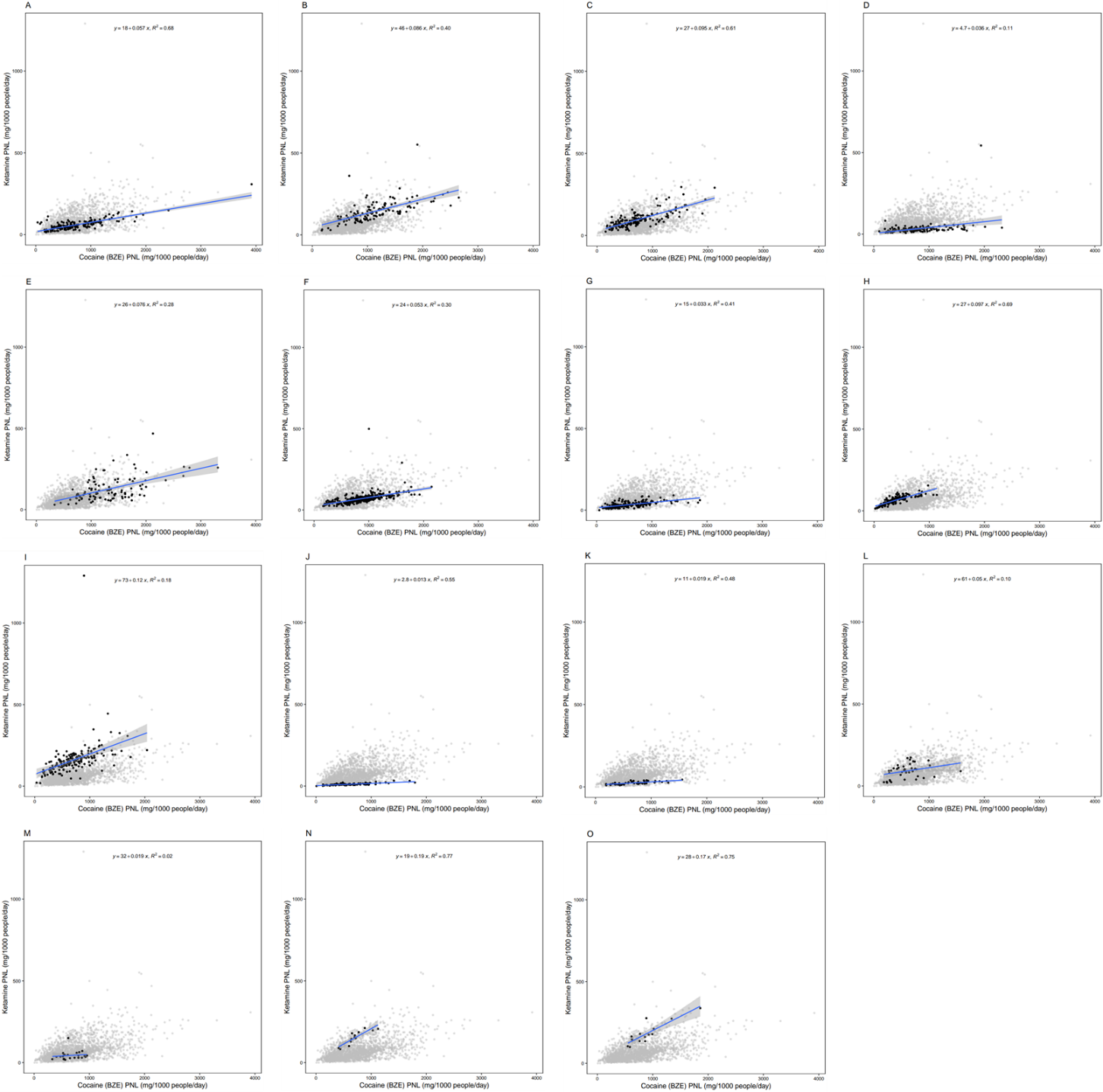
**

**Figure S9.** Correlation between cocaine (BZE) and ketamine estimated consumption data (mg/1000 people/day) for all sites A-O (highlighting each site dataset per draft) and showing the correlation coefficient (R^2^).


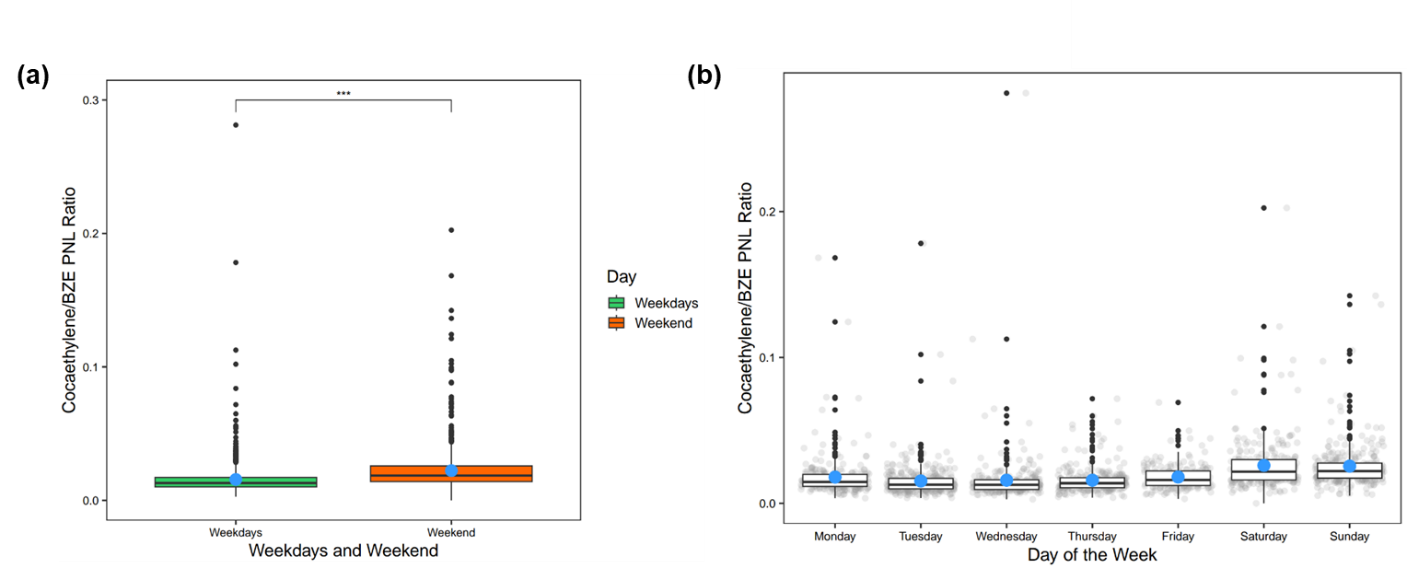


**Figure S10.** Box and whisker plot of the BZE/cocaethylene PNL ratio (mg/1000 people/day) across all WWTPs tested (n = 15) during (a) the weekdays (Tuesday to Thursday) and weekends (Friday to Monday) and (b) per day of the week. The blue dot represents the average value and *** means P ≤0.001.


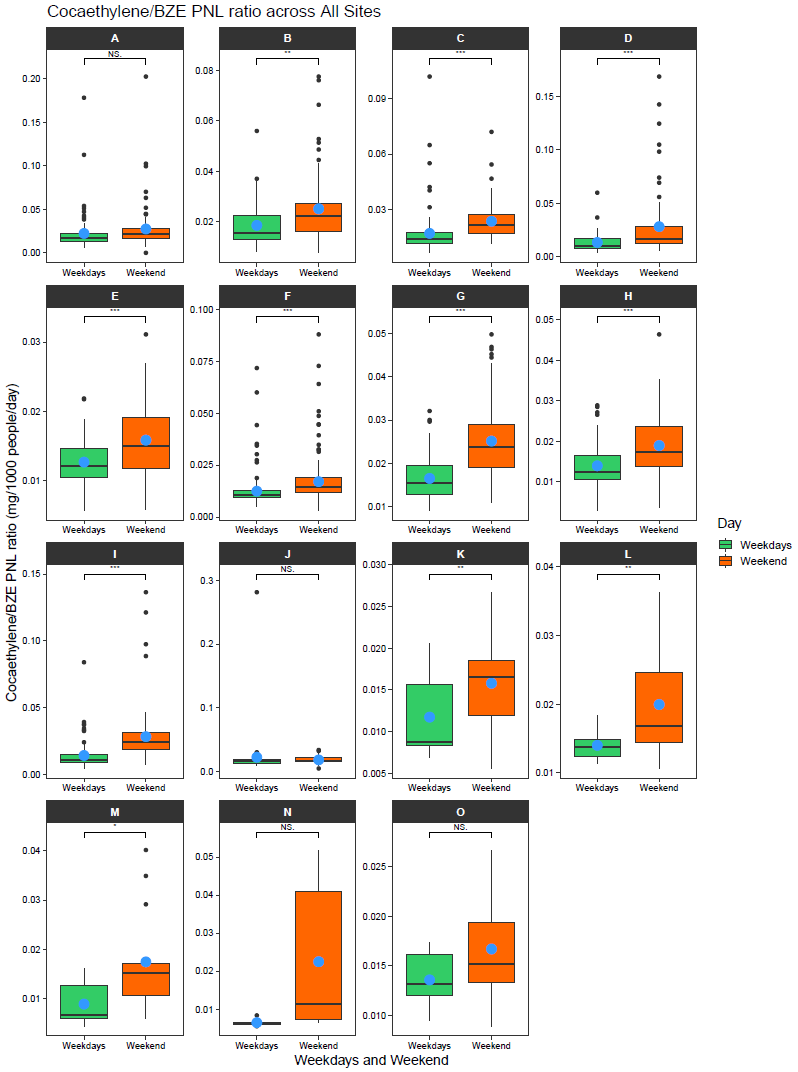


**Figure S11.** Box and whisker plots per sites of the cocaethylene/BZE PNL ratio (mg/1000 people/day) during the weekdays (Tuesday to Thursday) and weekends (Friday to Monday) across all sites A-O. The blue dot represents the average value and where NS. means not significant (P >0.05), * means P ≤0.05, ** means P ≤0.01 and *** means P ≤0.001.

**Table S8.** Number (%) of LSOAs and population coverage by each WWTP over the area covered by sampling sites and over all areas of England using 2021 UK Census Data (<https://www.nomisweb.co.uk/>).

| **Site Name** | **Number of LSOAs** | **% of LSOAs covered by the sampling WWTPs** (N=5,232 LSOAs) | **% of LSOAs covered by the sampling WWTPs compared to all LSOAs in England** (N=33,755 LSOAs) | **% of 2021 Census residential population covered by the sampling WWTPs**  (N=8,881,128 inhabitants) | **% of 2021 Census residential population covered by the sampling WWTPs over all areas of England** (N=56,554,891 inhabitants) |  |
| --- | --- | --- | --- | --- | --- | --- |
| Site A | 152 | 2.91 | 0.45 | 2.60 | 0.41 |  |
| Site B | 298 | 5.70 | 0.88 | 5.37 | 0.84 |  |
| Site C | 440 | 8.41 | 1.30 | 8.77 | 1.38 |  |
| Site D | 90 | 1.72 | 0.27 | 1.58 | 0.25 |  |
| Site E | 170 | 3.25 | 0.50 | 3.12 | 0.49 |  |
| Site F | 1,641 | 31.36 | 4.86 | 32.53 | 5.11 |  |
| Site G | 429 | 8.20 | 1.27 | 7.76 | 1.22 |  |
| Site H | 219 | 4.19 | 0.65 | 4.03 | 0.63 |  |
| Site I | 426 | 8.14 | 1.26 | 8.24 | 1.29 |  |
| Site J | | 83 | 1.59 | 0.25 | 1.57 | 0.25 |
| Site K | 394 | 7.53 | 1.17 | 7.73 | 1.21 |  |
| Site L | | 379 | 7.24 | 1.12 | 7.14 | 1.12 |
| Site M | 281 | 5.37 | 0.83 | 5.20 | 0.82 |  |
| Site N | 63 | 1.20 | 0.19 | 1.20 | 0.19 |  |
| Site O | 167 | 3.19 | 0.49 | 3.16 | 0.50 |  |
| All sites | 5,232 | 100.00 | 15.92 | 100.00 | 15.71 |  |

**Table S9.** Urban typology in areas with and without sampling WWTPs and by sampling WWTP in England.

|  | **Urban LSOAs number (%)** | **Rural LSOAs number (%)** |
| --- | --- | --- |
| **All areas in England** | **28,201 (83.55)** | **5,554 (16.45)** |
| **Areas not covered by sampling sites (n=1,419 WWTPs)** | **20,492 (81.39**) | **4,687 (18.61**) |
| **Areas covered by sampling sites (n=15 WWTPs** | **5,139 (98.22)** | **93 (1.78)** |
| Site A | 147 (96.71) | 5 (3.29) |
| Site B | 297 (99.66) | 1 (0.34) |
| Site C | 438 (99.55) | 2 (0.45) |
| Site D | 89 (98.89) | 1 (1.11) |
| Site E | 139 (81.76) | 31 (18.24) |
| Site F | 1640 (99.94) | 1 (0.06) |
| Site G | 405 (94.41) | 24 (5.59) |
| Site H | 213 (97.26) | 6 (2.74) |
| Site I | 419 (98.36) | 7 (1.64) |
| Site J | 81 (97.59) | 2 (2.41) |
| Site K | 393 (99.75) | 1 (0.25) |
| Site L | 373 (98.42) | 6 (1.58) |
| Site M | 276 (98.22) | 5 (1.78) |
| Site N | 62 (98.41) | 1 (1.59) |
| Site O | 167 (100) | 0 (0) |

**Table S10.** Descriptive statistics of daily illicit drug PNL values for each drug across all sampled WWTPs (n = 15) in 2022. In total, 1,746 samples were analysed, but only 1,730 samples had associated WWTP flow data to enable calculation of PNL.

| **Drug** | **N Samples** | **% Non-Missing Samples**  **Where PNL > 0** | **Mean PNL** | **Median PNL** | **IQR PNL** |
| --- | --- | --- | --- | --- | --- |
| 5-Aminoisotonitazene | 1730 | 99.08% | 0 | 0 | 0 |
| 6-Acetylcodeine | 1691 | 52.69% | 5.008 | 0 | 6.200 |
| 6-MAM | 555 | 4.47% | 4.908 | 3.320 | 4.689 |
| Amphetamine | 1730 | 56.53% | 68.429 | 0 | 97.666 |
| BZE | 1730 | 0% | 786.421 | 724.376 | 550.91 |
| Benzocaine | 1730 | 92.90% | 3.731 | 0 | 0 |
| Cocaethylene | 1730 | 0.06% | 13.919 | 10.529 | 10.860 |
| Cocaine | 1730 | 0% | 370.608 | 340.269 | 230.790 |
| EDDP | 1691 | 0.23% | 28.490 | 24.245 | 17.130 |
| Isotonitazene | 1691 | 96.79% | 0.001 | 0 | 0 |
| Ketamine | 1730 | 0.11% | 79.920 | 64.892 | 67.108 |
| Levamisole | 1730 | 64.03% | 8.746 | 0 | 3.967 |
| Lidocaine | 1730 | 0% | 30.793 | 26.548 | 19.537 |
| MDMA | 1730 | 31.79% | 21.853 | 12.112 | 26.357 |
| Methadone | 1730 | 0.17% | 13.620 | 11.394 | 7.688 |
| Methamphetamine | 1730 | 6.82% | 27.810 | 9 | 18.272 |
| Morphine | 1730 | 0.06% | 290.733 | 250.843 | 179.579 |
| Norketamine | 1730 | 80.47% | 9.502 | 0 | 0 |
| Phenacetin | 1730 | 97.54% | 1.015 | 0 | 0 |
| Procaine | 1722 | 93.41% | 0.875 | 0 | 0 |

**Table S11.** Associations between illicit drug PNLs in wastewater across England; effect estimates [95% confidence intervals] per 1 PNL increase from univariate linear-mixed effects models adjusting for WWTP as a random effect; * p < 0.05.

|  | **Independent Variable** | | | | | | | | | |
| --- | --- | --- | --- | --- | --- | --- | --- | --- | --- | --- |
| **Drug**  **(Dependent Variable) ↓** | **BZE** | **Cocaethylene** | **Cocaine** | **EDDP** | **Ketamine** | **Lidocaine** | **MDMA** | **Methadone** | **Methamphetamine** | **Morphine** |
| **BZE** |  | 24.942  [23.525, 26.359]* | 1.403  [1.326, 1.479]* | 17.054  [15.696, 18.411]* | 3.725  [3.419, 4.030]* | 3.849  [3.132, 4.567]* | 3.297  [2.897, 3.697]* | 23.049  [20.224, 25.874]* | 2.521  [1.881, 3.161]* | 1.630  [1.528, 1.731]* |
| **Cocaethylene** | 0.016  [0.015, 0.017]* |  | 0.035  [0.033, 0.037]* | 0.302  [0.265, 0.339]* | 0.073  [0.065, 0.081]* | 0.039  [0.020, 0.058]* | 0.088  [0.078, 0.098]* | 0.597  [0.525, 0.668]* | 0.039  [0.023, 0.055]* | 0.021  [0.018, 0.024]* |
| **Cocaine** | 0.307  [0.290, 0.323]* | 11.713  [11.054, 12.373]* |  | 8.083  [7.435, 8.731]* | 1.503  [1.354, 1.652]* | 1.703  [1.367, 2.040]* | 1.002  [0.807, 1.197]* | 11.842  [10.533, 13.152]* | 1.262  [0.964, 1.560]* | 0.621  [0.569, 0.673]* |
| **EDDP** | 0.015  [0.014, 0.016]* | 0.421  [0.369, 0.473]* | 0.032  [0.030, 0.035]* |  | 0.077  [0.067, 0.086]* | 0.133  [0.112, 0.154]* | 0.038  [0.026, 0.051]* | 1.699  [1.629, 1.769]* | 0.055  [0.035, 0.074]* | 0.042  [0.039, 0.045]* |
| **Ketamine** | 0.066  [0.061, 0.072]* | 2.001  [1.775, 2.227]* | 0.123  [0.111, 0.135]* | 1.588  [1.385, 1.792]* |  | 0.453  [0.357, 0.549]* | 0.430  [0.377, 0.483]* | 2.327  [1.933, 2.721]* | 0.293  [0.206, 0.380]* | 0.142  [0.126, 0.158]* |
| **Lidocaine** | 0.016  [0.013, 0.019]* | 0.240  [0.123, 0.357]* | 0.032  [0.026, 0.038]* | 0.643  [0.546, 0.739]* | 0.103  [0.082, 0.125]* |  | 0.025  [-0.002, 0.053] | 1.160  [0.976, 1.343]* | 0.088  [0.047, 0.129]* | 0.051  [0.044, 0.059]* |
| **MDMA** | 0.039  [0.035, 0.044]* | 1.607  [1.420, 1.794]* | 0.055  [0.044, 0.066]* | 0.504  [0.331, 0.676]* | 0.284  [0.248, 0.320]* | 0.078  [-0.002, 0.159] |  | 0.534  [0.207, 0.861]* | 0.150  [0.083, 0.218]* | 0.043  [0.029, 0.057]* |
| **Methadone** | 0.005  [0.005, 0.006]* | 0.216  [0.189, 0.242]* | 0.013  [0.011, 0.014]* | 0.336  [0.322, 0.350]* | 0.031  [0.026, 0.036]* | 0.066  [0.055, 0.076]* | 0.011  [0.005, 0.018]* |  | 0.023  [0.013, 0.033]* | 0.018  [0.016, 0.020]* |
| **Methamphetamine** | 0.013  [0.010, 0.016]* | 0.322  [0.191, 0.452]* | 0.029  [0.022, 0.036]* | 0.327  [0.211, 0.442]* | 0.084  [0.059, 0.109]* | 0.113  [0.060, 0.165]* | 0.061  [0.031, 0.092]* | 0.509  [0.291, 0.727]* |  | 0.035  [0.026, 0.044]* |
| **Morphine** | 0.222  [0.208, 0.236]* | 4.358  [3.711, 5.005]* | 0.388  [0.355, 0.420]* | 6.647  [6.142, 7.152]* | 1.084  [0.964, 1.204]* | 1.713  [1.453, 1.974]* | 0.504  [0.347, 0.660]* | 10.552  [9.539, 11.566]* | 0.927  [0.689, 1.166]* |  |

**Table S12.** Pairwise number of valid daily illicit drug PNL values; total number of paired PNL values for a given day at any of the sampled WWTPs (n = 15).

|  | **BZE** |  |  |  |  |  |  |  |  |
| --- | --- | --- | --- | --- | --- | --- | --- | --- | --- |
| **Cocaethylene** | 1730 | **Cocaethylene** |  |  |  |  |  |  |  |
| **Cocaine** | 1730 | 1730 | **Cocaine** |  |  |  |  |  |  |
| **EDDP** | 1691 | 1691 | 1691 | **EDDP** |  |  |  |  |  |
| **Ketamine** | 1730 | 1730 | 1730 | 1691 | **Ketamine** |  |  |  |  |
| **Lidocaine** | 1730 | 1730 | 1730 | 1691 | 1730 | **Lidocaine** |  |  |  |
| **MDMA** | 1730 | 1730 | 1730 | 1691 | 1730 | 1730 | **MDMA** |  |  |
| **Methadone** | 1730 | 1730 | 1730 | 1691 | 1730 | 1730 | 1730 | **Methadone** |  |
| **Methamphetamine** | 1730 | 1730 | 1730 | 1691 | 1730 | 1730 | 1730 | 1730 | **Methamphetamine** |
| **Morphine** | 1730 | 1730 | 1730 | 1691 | 1730 | 1730 | 1730 | 1730 | 1730 |

**
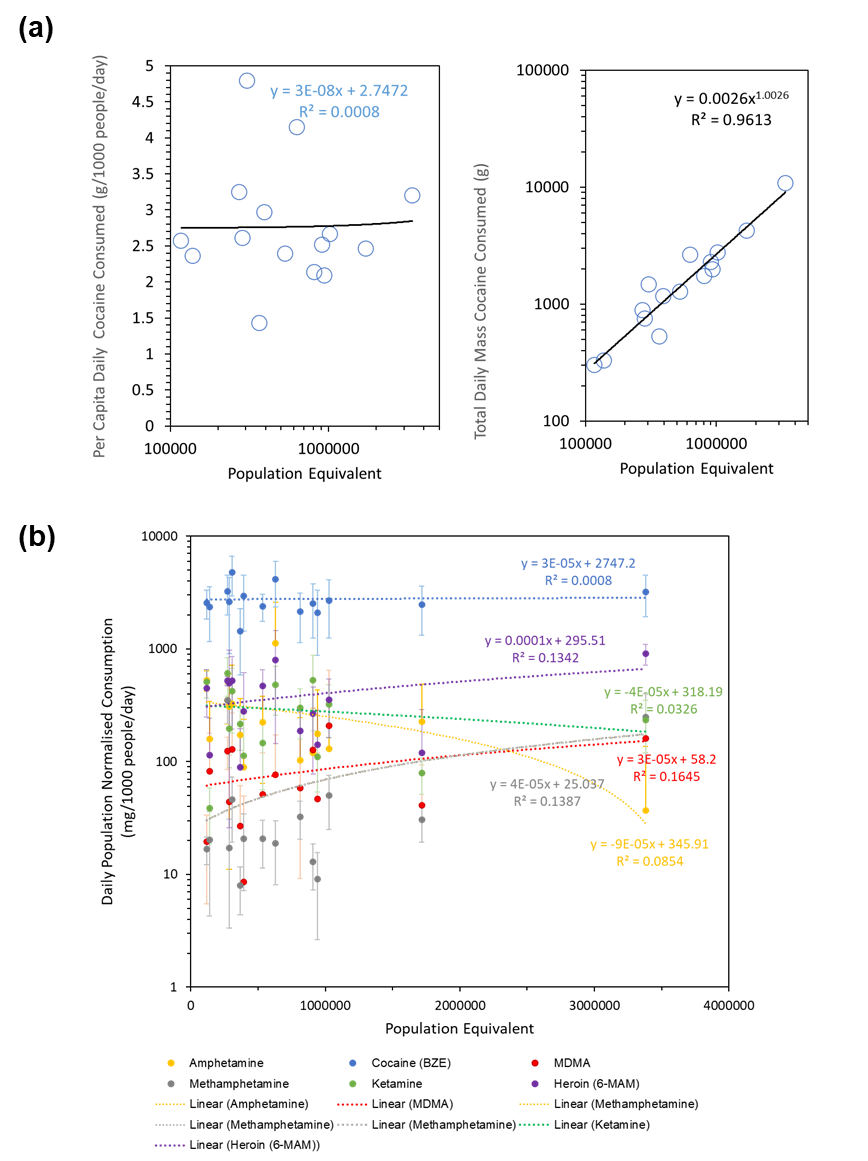
**

**Figure S12.** (a) Daily cocaine consumption across sites, shown both as per capita values (left) and as total mass (right). (b) Corresponding plot of per capita daily consumption (log scale) and population equivalent (PE) for all six drugs.

**Table S13.** Wilcoxon rank-sum test results comparing average estimated consumption levels (mg/1000 people/day) between sites in the North and South of England. Columns show the drug name, the two groups compared (North vs South), the number of sites per group (n1 and n2), the test statistic (W), the unadjusted p-value, the p-value adjusted for multiple comparisons using the Benjamini-Hochberg false discovery rate (p.adj), and the corresponding significance level (p.adj.signif; ns = not significant, * = p ≤ 0.05, ** = p ≤ 0.01, *** = p ≤ 0.001).

| **Drug** | **Group 1** | **Group 2** | **n1** | **n2** | **Statistic** | **p** | **p.adj** | **p.adj.signif** |
| --- | --- | --- | --- | --- | --- | --- | --- | --- |
| Amphetamine | North | South | 9 | 6 | 32 | 0.596 | 0.816 | ns |
| Cocaine | North | South | 9 | 6 | 23 | 0.68 | 0.816 | ns |
| Heroin | North | South | 9 | 6 | 26 | 0.953 | 0.953 | ns |
| Ketamine | North | South | 9 | 6 | 19 | 0.377 | 0.816 | ns |
| MDMA | North | South | 9 | 6 | 12 | 0.875 | 0.525 | ns |
| Methamphetamine | North | South | 9 | 6 | 23 | 0.68 | 0.816 | ns |

**References**

1. ICH. Validation of analytical procedures: text and methodology Q2(R1). 2005.

2. Baker DR, Kasprzyk-Hordern B. Multi-residue analysis of drugs of abuse in wastewater and surface water by solid-phase extraction and liquid chromatography–positive electrospray ionisation tandem mass spectrometry. Journal of Chromatography A. 2011 Mar;1218(12):1620–31.
